# High-Grade Glioma Treatment Response Monitoring Biomarkers: A Position Statement on the Evidence Supporting the Use of Advanced MRI Techniques in the Clinic, and the Latest Bench-to-Bedside Developments. Part 1: Perfusion and Diffusion Techniques

**DOI:** 10.3389/fonc.2022.810263

**Published:** 2022-03-03

**Authors:** Otto M. Henriksen, María del Mar Álvarez-Torres, Patricia Figueiredo, Gilbert Hangel, Vera C. Keil, Ruben E. Nechifor, Frank Riemer, Kathleen M. Schmainda, Esther A. H. Warnert, Evita C. Wiegers, Thomas C. Booth

**Affiliations:** ^1^Department of Clinical Physiology, Nuclear Medicine and PET, Copenhagen University Hospital Rigshospitalet, Copenhagen, Denmark; ^2^Biomedical Data Science Laboratory, ITACA, Universitat Politècnica de València, Valencia, Spain; ^3^Department of Bioengineering and Institute for Systems and Robotics-Lisboa, Instituto Superior Técnico, Universidade de Lisboa, Lisbon, Portugal; ^4^Department of Neurosurgery, Medical University, Vienna, Austria; ^5^High-Field MR Centre, Department of Biomedical Imaging and Image-Guided Therapy, Medical University, Vienna, Austria; ^6^Department of Radiology and Nuclear Medicine, Amsterdam UMC, Amsterdam, Netherlands; ^7^International Institute for the Advanced Studies of Psychotherapy and Applied Mental Health, Department of Clinical Psychology and Psychotherapy, Babes-Bolyai University, Cluj-Napoca, Romania; ^8^Mohn Medical Imaging and Visualization Centre (MMIV), Department of Radiology, Haukeland University Hospital, Bergen, Norway; ^9^Department of Biophysics, Medical College of Wisconsin, Milwaukee, WI, United States; ^10^Department of Radiology & Nuclear Medicine, Erasmus MC, Rotterdam, Netherlands; ^11^Department of Radiology, University Medical Center Utrecht, Utrecht, Netherlands; ^12^ School of Biomedical Engineering and Imaging Sciences, St. Thomas’ Hospital, King’s College London, London, United Kingdom; ^13^Department of Neuroradiology, King’s College Hospital NHS Foundation Trust, London, United Kingdom

**Keywords:** magnetic resonance imaging, glioma, perfusion, diffusion, pseudoprogression, monitoring biomarkers, glioblastoma, high-grade glioma

## Abstract

**Objective:**

Summarize evidence for use of advanced MRI techniques as monitoring biomarkers in the clinic, and highlight the latest bench-to-bedside developments.

**Methods:**

Experts in advanced MRI techniques applied to high-grade glioma treatment response assessment convened through a European framework. Current evidence regarding the potential for monitoring biomarkers in adult high-grade glioma is reviewed, and individual modalities of perfusion, permeability, and microstructure imaging are discussed (in Part 1 of two). In Part 2, we discuss modalities related to metabolism and/or chemical composition, appraise the clinic readiness of the individual modalities, and consider post-processing methodologies involving the combination of MRI approaches (multiparametric imaging) or machine learning (radiomics).

**Results:**

High-grade glioma vasculature exhibits increased perfusion, blood volume, and permeability compared with normal brain tissue. Measures of cerebral blood volume derived from dynamic susceptibility contrast-enhanced MRI have consistently provided information about brain tumor growth and response to treatment; it is the most clinically validated advanced technique. Clinical studies have proven the potential of dynamic contrast-enhanced MRI for distinguishing post-treatment related effects from recurrence, but the optimal acquisition protocol, mode of analysis, parameter of highest diagnostic value, and optimal cut-off points remain to be established. Arterial spin labeling techniques do not require the injection of a contrast agent, and repeated measurements of cerebral blood flow can be performed. The absence of potential gadolinium deposition effects allows widespread use in pediatric patients and those with impaired renal function. More data are necessary to establish clinical validity as monitoring biomarkers. Diffusion-weighted imaging, apparent diffusion coefficient analysis, diffusion tensor or kurtosis imaging, intravoxel incoherent motion, and other microstructural modeling approaches also allow treatment response assessment; more robust data are required to validate these alone or when applied to post-processing methodologies.

**Conclusion:**

Considerable progress has been made in the development of these monitoring biomarkers. Many techniques are in their infancy, whereas others have generated a larger body of evidence for clinical application.

## 1 Introduction

High-grade gliomas (adult-type diffuse WHO grade 3 and 4 gliomas) account for up to 85% of all new cases of malignant primary brain tumors diagnosed every year, with an incidence of approximately 5/100,000 person years in Europe and North America (1). Of these, approximately 70% are either “glioblastomas, isocitrate dehydrogenase (IDH)-wildtype” or “astrocytoma, IDH-mutant, grade 4” (2). Approximately 15% are “astrocytoma, IDH-mutant, grade 3” and approximately 10% are “oligodendroglioma, IDH-mutant and (chromosome) 1p/19q deleted, grade 3.” Malignant primary brain tumors cause the greatest number of years of life lost than any other cancer (3). Grade 4 glioma is particularly devastating: The median survival without any treatment is less than six months and with standard-of-care treatment is only 14.6 months (4). In adults aged up to 70 years with good performance status, maximal safe tumor resection followed by radiotherapy with concomitant and adjuvant temozolomide has been recommended as the standard-of-care treatment for grade 4 glioma since 2005 (4–6).

During and after treatment, “monitoring biomarkers” are measured serially and are required to detect any change in the extent of glioma infiltration or provide evidence of treatment response (7). Magnetic resonance imaging (MRI) is particularly useful in determining treatment response as it is noninvasive and captures the entire tumor volume as well as adjacent tissues. Furthermore, in nearly all high-grade gliomas, the integrity of the blood-brain barrier (BBB) is disrupted. Following intravenous administration of gadolinium-based contrast agents (GBCA), the hydrophilic contrast molecules diffuse out of the vessel lumen and accumulate within the extravascular extracellular space, manifesting as contrast-enhancing hyperintense regions on *T*_1_-weighted sequences (8). Subsequently, MRI has been incorporated into recommendations for determining treatment response in clinical trials (9). In these recommendations, treatment response assessment is based on simple linear metrics of contrast-enhancing tumor, specifically, the product of the maximal perpendicular cross-sectional dimensions (in “measurable” lesions, which are defined as > 10 mm in all perpendicular dimensions). The recommendations are based on expert opinion informed by observational studies and derived from the biologically plausible assumption that changes in tumor size identify progression of disease, potentially before it becomes clinically apparent, resulting in a lead time improvement for therapeutic intervention (10). Indeed, there may be benefits in changing management before the development of irreversible disability or before the extent of tumor precludes intervention. Some justification for enhancement as a disease proxy has been inferred from data showing that enhancing tumor size and extent of resection are “prognostic biomarkers” (7) at both first presentation and recurrence (11, 12).

The trial assessment recommendations, incorporated in a less stringent form during routine clinical assessment (13), allow for an early change in treatment strategy, for example, termination of ineffective treatments or switching to second-line therapies (14). However, there are four important challenges using conventional structural MRI protocols.

First, there is a paucity of evidence that earlier diagnosis of disease recurrence using conventional structural MRI influences prognosis (10). It is noted that individual enhancing tumor growth trajectories vary between individuals with the same histological tumor type.

Second, seemingly simple measurements can still be challenging because tumors have a variety of shapes, tumors may be confined to a cavity rim, and the edge of tumors may be difficult to define (15). For example, large, cyst-like gliomas are common and are often “non-measurable” unless a solid peripheral nodular component fulfils the above “measurable” criteria.

**Figure 1:**
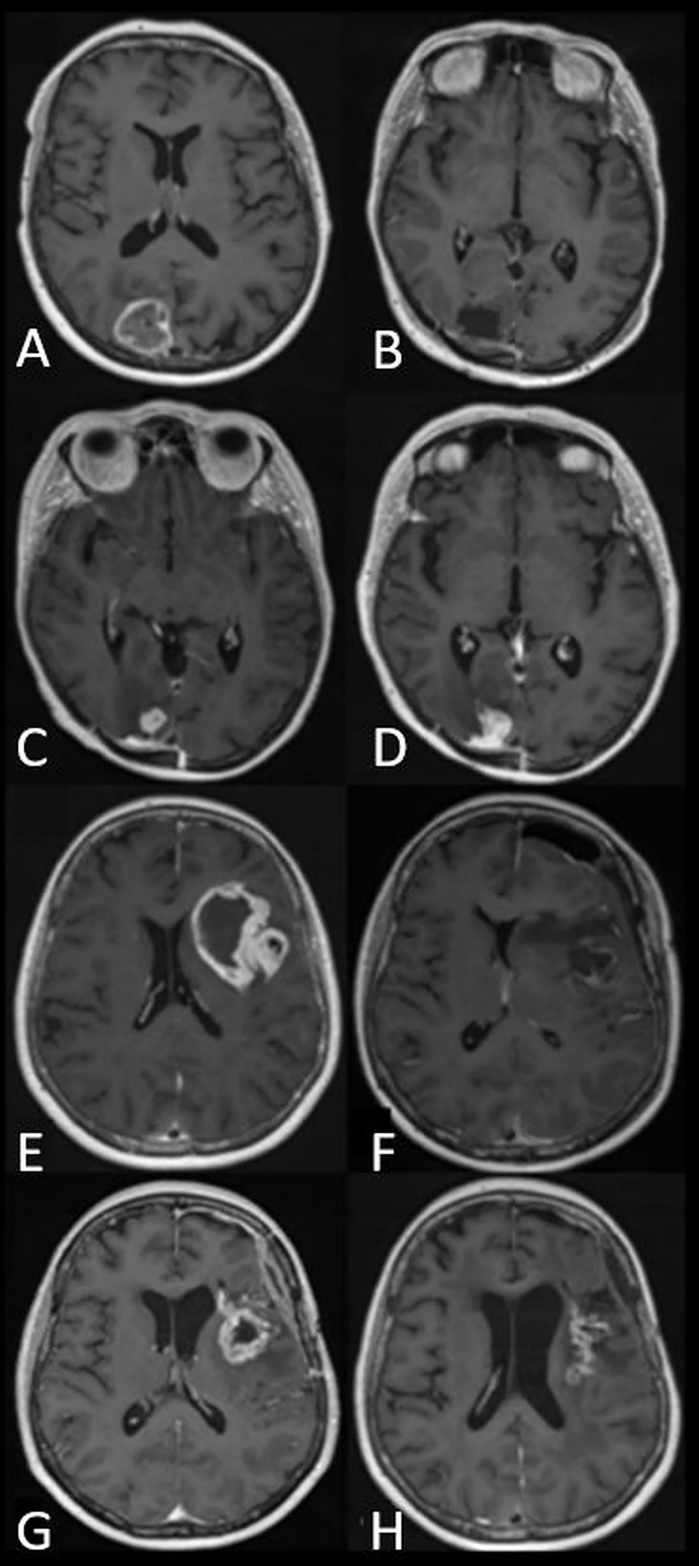
Panels of axial *T*_1_-weighted images after contrast administration in two patients with glioblastoma, IDH-wildtype. **(A–D)** demonstrate tumor progression. **(A)** Preoperative appearance of an occipital glioblastoma. **(B)** MRI performed five days after resection with no enhancement suggestive of residual tumor. **(C)** The patient continued with standard-of-care radiotherapy and temozolomide. Within three months of radiotherapy, a new enhancing lesion could be seen at the margin of the postoperative cavity. **(D)** The enhancing lesion had increased in size three months later and was confirmed to represent tumor recurrence after repeat surgery. **(E–H)** demonstrate pseudoprogression. **(E)** Preoperative appearance of an insular glioblastoma. **(F)** Postoperative appearance 24 hours after surgery showing blood degradation products, with no enhancement suggestive of residual tumor. **(G)** The patient continued with standard-of-care radiotherapy and temozolomide. MRI performed within six months of radiotherapy demonstrated a new, contrast-enhancing lesion. **(H)** Follow-up MRI at monthly intervals showed a gradual decrease in the size of the contrast-enhancing lesion without a change to standard-of-care temozolomide or corticosteroid use. The image shown here is the MRI performed four months later.

Third, there is a lack of biological specificity for contrast enhancement, which can lead to false positive, false negative, and indeterminate results, particularly relating to post-treatment related pseudophenomena in glioma (10). In high-grade glioma, pseudoprogression is an early post-treatment-related effect typically occurring within six months of finishing concomitant temozolomide and radiotherapy (**Figure 1**), whereas pseudoresponse typically occurs after anti-angiogenic agents such as bevacizumab have been administered. False positive progression and false negative treatment response are manifest as an increase or decrease in MRI contrast-enhancing volume, respectively. Delayed treatment effects, such as increased enhancement due to radiation necrosis, can similarly cause false positive progression. Other examples of non-specificity include post-operative peritumoral parenchymal enhancement following operative “tissue handling” or following operative infarction.

Fourth, due to the non-specificity of changes in contrast enhancing volume, treatment response assessment that is typically made in a retrospective manner as confirmatory imaging, is required to demonstrate a sustained increase (**Figure 1**) or a sustained decrease in enhancing volume. This leads to a delay in diagnosis.

**Figure 2:**
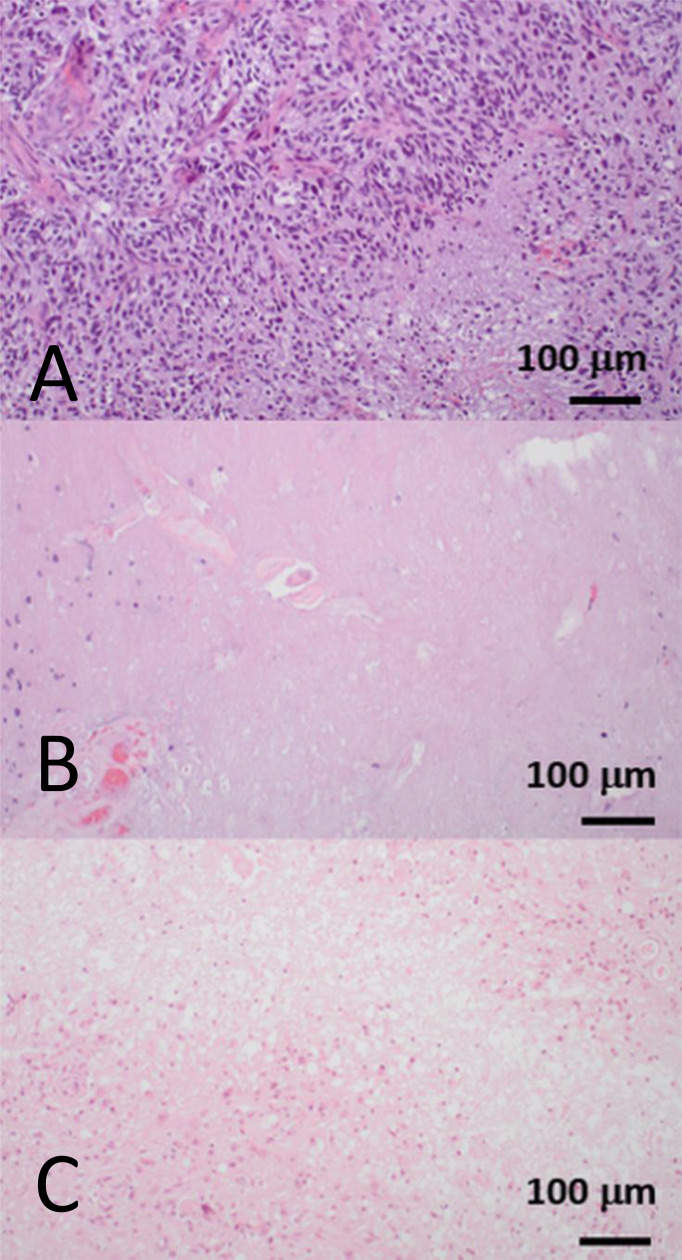
Hematoxylin and eosin-stained biopsy samples showing three distinct histopathological patterns from three patients who were treated for glioblastoma, IDH-wildtype. All biopsies were obtained from a contrast-enhancing region that had increased in size on serial *T*_1_-weighted images during follow-up imaging. **(A)** A fully viable tumor recurrence with dense cellularity and pseudopalisading necrosis. This corresponds to tumor progression. **(B)** Depopulated tumor with necrotizing treatment effect. This corresponds to the radiological appearances of pseudoprogression. **(C)** Nearly absent tumor cell compartment with extensive necrotizing treatment effect and hyalinizing vasculopathy. This corresponds to the delayed treatment effect, radiation necrosis. Pseudoprogression and radiation necrosis are two well-documented forms of “post-treatment related effect.” Pseudoprogression generally occurs within the six months following completion of chemoradiotherapy, and resolves or stabilizes without additional treatment, whereas radiation necrosis generally occurs beyond six months, up to several years after radiotherapy, and is often more severe and progressive (16–19). Images courtesy of Dr. J Huse, Department of Pathology, MD Anderson Cancer Center, United States.

Clearly, contemporaneous, accurate, and reliable monitoring biomarkers are required for high-grade glioma treatment response assessment. Due to the non-specific nature of contrast enhancement, histopathology is sometimes employed as a combined or alternative monitoring biomarker; often it is considered the reference standard of treatment response (**Figure 2**). However, there are several challenges when using histopathology as a monitoring biomarker. First, there is a paucity of evidence that earlier diagnosis of viable tumor using biopsy influences prognosis (2). Second, repeat tissue sampling has a high risk of procedure-related morbidity in patients with high-grade glioma compared to other systemic cancers (20). Third, biopsy has a potential drawback of sampling bias (21). Fourth, there is a need to improve and better systematize the application of histological and molecular analysis to diffuse glioma in the recurrent setting; currently, it is not standardized, causing a variety of inter-observer diagnostic interpretations given the background of extensive post-therapy-related changes (2).

An emerging alternative approach is to harness the potential value of circulating biomarkers (including circulating tumor cells, exosomes, and microRNAs) to monitor disease progression in glioma patients (22). For example, a recent study found that serum microRNA levels did not increase in cases of pseudoprogression, and increased levels were associated with tumor progression (23). However, as with any potential monitoring blood or cerebral spinal fluid biomarker, potential use requires further evaluation and validation in larger scale prospective studies before implementation into routine clinical practice can be envisaged.

Another promising approach, which concords with an impetus to derive an evidence-base for follow-up imaging of high-grade gliomas driven both by researchers as well as patients and caregivers (24–26), is to use advanced imaging techniques. Considerable technical developments have occurred in the last three decades; however, clinical translation is far from ubiquitous. A European survey in 2016 suggested dynamic susceptibility contrast-enhanced (DSC) MRI and ^1^H-magnetic resonance spectroscopy was used in the clinic routinely in 82% and 80% of hospitals respectively (27). However, with only 3% survey response rate, it is unlikely to be representative of European practice. A 2020 survey of all UK neuro-oncology centers with 100% response rate from all three lead specialists (neuroradiology, neuro-oncology, and neurosurgery) within all centers showed that only 10% of centers routinely used any advanced MRI technique and a third of centers used such techniques in selected cases (13). It is noteworthy that many respondents suggested that advanced imaging techniques would improve their practice.

The purpose of this position statement is to summarize the evidence for the use of advanced MRI techniques as monitoring biomarkers in adult high-grade glioma in the clinic, and to highlight the latest bench-to-bedside developments.

## 2 Materials and Methods

Clinicians, engineers, and physicists with expertise in advanced MRI techniques applied to high-grade glioma, convened virtually through the European Cooperation in Science and Technology (COST) Glioma MR Imaging 2.0 (GliMR) initiative (28) from July 2020 through July 2021, which also included experts from outside of Europe. A working group analyzed the available evidence for potential high-grade glioma monitoring biomarkers derived from advanced imaging techniques. The consensus decision was to focus on monitoring biomarkers that can reliably differentiate post-treatment-related effect from true tumor progression during (or before) the point when contrast enhancement on *T*_1_-weighted MRI images first increases. False-negative progression (pseudoresponse) during second line treatment is a concern in the United States but rarely in Europe, as the European Medicines Agency concluded that progression-free survival bevacizumab trial outcome measures are inherently confounded and the use of bevacizumab is not supported (29). Therefore, pseudoresponse was not the focus of the position statement.

Potential biomarkers were derived predominantly from the individual image acquisition modalities of perfusion and/or permeability (using DSC MRI techniques, dynamic contrast enhanced [DCE] and arterial spin labeling [ASL]), microstructure (diffusion MRI techniques), metabolism and/or chemical composition (magnetic resonance spectroscopy and chemical exchange saturation transfer [CEST] technique, and MRI combined with positron emission tomography [PET]). We also considered post-processing methodologies involving the combination of MRI approaches (multiparametric imaging) and image feature analysis techniques using machine learning (radiomics).

We agreed the work would be a *position statement* (30) promoting discussion on emerging topics and indicate the evidence gaps, strengths and limitations. Together this would provide the foundation for producing a multi-stakeholder downstream *guideline* (30) beyond the current position statement.

Specifically, it was agreed that advanced imaging technique analyses would

- summarize methodology,
- highlight strengths and weaknesses,
- determine clinical diagnostic accuracy, and
- expound the state-of-the-art and future developments.

Finally, we assessed the incorporation into national and/or international technical and/or clinical guidelines and indicated the current level of development and clinical readiness (presented in Part 2).

Advanced imaging technique analyses were compiled by subject matter experts and incorporated into a manuscript and circulated to the working group members. Edits and feedback were incorporated until all authors were in agreement with the content, and a position statement was produced summarizing the evidence for the use of advanced MRI techniques as monitoring biomarkers in the clinic, and highlighting the latest bench-to-bedside developments.

To determine clinical diagnostic accuracy, we performed MEDLINE (including PubMed), Embase and Cochrane Register searches for recent systematic reviews and meta-analyses, favoring those which followed Preferred Reporting Items for Systematic Reviews and Meta-Analysis: Diagnostic Test Accuracy (PRISMA-DTA) methodology (31). We also performed searches to analyze individual clinical studies related to each advanced imaging technique since the time of the included systematic review; if a systematic review was published before 2015, we confined our searches to 2015–2021. Search terms are listed in **Supplementary Table S1**. Given that the position statement describes a broad range of studies involving several imaging approaches (a range of MRI advanced techniques, PET, and post-processing methodologies) and several target conditions (pseudoprogression, radiation necrosis, or a combination of both) a PRISMA-DTA analysis addressing a specific question on diagnostic accuracy was beyond our remit. Nonetheless, components of the PRISMA-DTA methodology have been incorporated where practicable.

## 3 Results

### 3.1 An Overview of Perfusion and Permeability

A hallmark of cancers is angiogenesis, the formation of new tumor vessels to sustain metabolic demands of the growing tumor (32). Its main trigger is hypoxia *via* HIF-1 (hypoxia inducible factor 1) release and cascades of other pro-angiogenic factors, e.g., vascular endothelial growth factor. Compared to normal vessels, tumor vessels are larger, more irregular, and, importantly, highly permeable. The increased permeability leads to a disrupted BBB, allowing the extravasation of macromolecules (such as contrast agents) across the capillary wall (33). The glioma vasculature will thus exhibit increased perfusion, blood volume, and permeability compared with normal brain tissue.

Although the exact definition may depend on the method applied (34), perfusion is generally defined as the passage of blood through the tissue microcirculation. In the brain, cerebral blood flow (CBF) is traditionally used synonymously with perfusion, and both are measured in units of volume of blood per volume or weight of tissue per time (ml/100 ml/min or ml/100 mg/min). On the other hand, the term perfusion imaging is often used in a wider sense and also includes other aspects of the vascular function of an organ or tissue such as blood volume or vessel permeability. In the brain, the most commonly measured perfusion parameters are CBF and cerebral blood volume (CBV). In order to distinguish perfusion parameter maps (showing regional distribution within the brain) from global brain measurements, in imaging they are sometimes referred to as regional CBF and CBV (rCBF and rCBV, respectively). In a possibly confusing fashion, measures of CBF and CBV that are obtained voxelwise *relative* to the rest of the brain, but not in absolute units, are also often referred to as rCBF and rCBV, respectively. The latter definition of rCBF/rCBV is commonly used in the context of the perfusion imaging techniques described here. For this reason, we chose to use CBF and CBV to refer to absolute measurements of perfusion and blood volume, while keeping rCBF and rCBV for relative versions of these parameters. Moreover, perfusion imaging measurements of brain tumors are commonly normalized to a reference, for example healthy appearing brain of the contralateral hemisphere. In this case, normalized values are referred to as nRCBF/nRCBV, respectively. Another perfusion related parameter is vessel permeability, which may be quantified in terms of a transfer constant (K^trans^) or a permeability surface area product (PS), depending on the measurement technique and associated modeling approach.

DSC and DCE techniques are based on dynamic MR imaging during the bolus passage of a GBCA to provide images of perfusion- and permeability-related parameters. DSC is a *T*_2_/*T*_2_* method most often used to assess vascular proliferation in terms of CBV. Conversely, DCE is a *T*_1_-weighted method, which in its most widely used form provides information on a combination of tissue perfusion and vessel permeability as measured by K^trans^ in addition to plasma volume (Vp). Alternatively, arterial spin labeling (ASL) is a completely noninvasive and potentially quantitative technique without GBCA, which is based on labeling arterial water in order to provide an endogenous, freely diffusible perfusion tracer (35). ASL provides perfusion-weighted images, and it can be used to quantify tissue perfusion (CBF).

### 3.2 DSC-MRI

#### 3.2.1 Methodology

**Figure 3:**
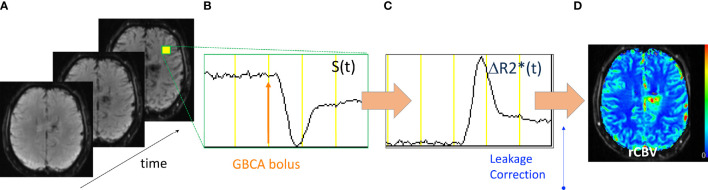
Acquisition and processing of DSC-MRI data. **(A)** GRE-EPI images are collected with high temporal resolution. **(B)** The signal time course (S(t)) from a representative voxel shows the collection of baseline signal then the bolus injection of GBCA after which there is a transient decrease in S(t) and return toward baseline. **(C)** The S(t) is converted into the change in the *T*_2_* relaxation rate (ΔR2*(t)) from which **(D)** leakage-correction algorithms are applied and rCBV maps created.

With DSC-MRI, *T*_2_- or *T*_2_*-weighted images are acquired with high temporal resolution during the bolus administration of a GBCA (**Figure 3**). The bolus passage induces a gradient of susceptibility between the intravascular and extravascular tissue compartments causing a transient decrease in the signal intensity (36). Most often *T*_2_*-weighted gradient-echo (GRE) echo planar imaging (EPI) methods are used, which are sensitive to vessels of all diameters (37). *T*_2_-weighted images, obtained with spin-echo (SE) EPI methods, provide CBV maps primarily sensitive to microvessels. Subsequent descriptions will be given for GRE-based methods unless otherwise noted. The same basic principles for data acquisition and processing apply for SE-based methods.

As further illustrated in **Figure 3**, the DSC signal time course, S(t) is converted to a change in the *T*_2_* relaxation rate (ΔR2*(t)),


Equation 1 $$\Delta R2^{*}(t)=\frac{-1}{TE}\ln\left(\frac{S(t)}{S_{b}}\right),$$

where S_b_ is the mean of the baseline (pre-bolus) signal intensity whose integration provides an estimate of the relative cerebral blood volume (rCBV).


Equation 2 $$rCBV=\int \Delta R2^{*}(t)dt$$

This measure is termed “relative” because it provides a voxelwise value *relative* to the rest of the brain.

Alternatively, an absolute measure of CBV can be determined from the ratio of the tissue (C_t_(t)) (which is assumed equivalent to ΔR2*(t)) and arterial concentration-time curve (C_a_(t)) first-pass areas,


Equation 3 $$CBV=\kappa \frac{\int C_{t}(\tau )d\tau }{\int C_{a}(\tau )d\tau },$$

where κ is the scale factor accounting for the density of brain tissue and the differences in hematocrit between capillaries and large vessels (38). As indicated, an absolute measure of CBV requires the determination of Ca(t), also referred to as the arterial input function (AIF). More often, for purposes of comparison, the rCBV will be normalized by dividing the rCBV in each image voxel by the mean rCBV determined from a reference tissue region of interest (ROI), such as normal appearing white matter giving normalized rCBV (nRCBV) values. Like absolute CBV, the nRCBV maps provide greater consistency for the comparison of rCBV across time and patients. Note that in some instances the term nRCBV may be used interchangeably with normalized CBV (nCBV), with the implicit assumption that the CBV is relative. In all cases, an explicit description of the steps used to create CBV should be provided, with clear definitions of nomenclature provided. Finally, another newer approach termed “standardization” has been devised to calibrate the rCBV maps directly (39) without requiring a normalizing ROI. Standardized rCBV (sRCBV) has demonstrated greater consistency across time (39) and improved repeatability compared with nRCBV (40). Both nRCBV and sRCBV have been used with success in several brain tumor clinical trials (41–43).

#### 3.2.2 Correction for Leakage Effects

A key assumption of DSC-MRI is that GBCA remains compartmentalized within the intravascular space, causing a susceptibility difference between the intravascular and extravascular compartments. However, with brain tumors, which often have a disrupted BBB, this assumption is violated. GBCA extravasation causes changes to the extravascular *T*_1_ and *T*_2_ (GBCA causes a change in the relaxation rate of the local tissue, which is inversely proportional to *T*_1_ and *T*_2_ [*R*_1_ = 1/*T*_1_; *R*_2_ = 1/*T*_2_]) in ways that can profoundly affect the DSC-MRI time course (44) and, thus, the accuracy of the rCBV estimate (45, 46). Therefore, different MRI acquisition and post-processing approaches have been used to diminish and/or correct for confounding GBCA leakage effects. One common approach is to administer a single-dose preload of GBCA before the collection of the DSC-MRI data, during which a second dose of GBCA is administered. The preload serves to diminish the effects of contrast extravasation that would occur during the DSC-MRI acquisition (45). One study demonstrated that use of a single-dose preload (0.1 mmol Gd/kg) with a 5-minute incubation time before the DSC-MRI dose, provided the best distinction in rCBV between tumor and treatment effect (47). Yet, even with a contrast agent preload, a correlation with tumor grade was only evident when a post-processing correction for leakage effects was also applied (46). The Boxerman-Schmainda-Weisskoff method (48) has become one of the most widely used leakage correction methods, but several modifications have since been proposed that deserve further consideration (49–51).

**Figure 4:**
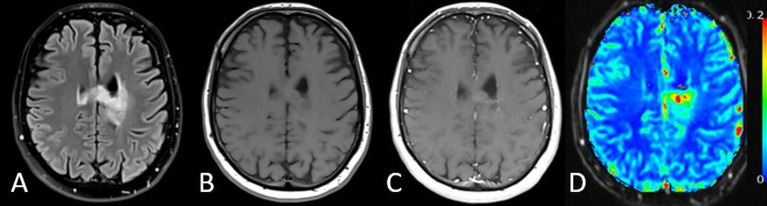
MRI results from a 32-year-old patient with a grade 2 oligodendroglioma. **(A)** *T*_2_-weighted FLAIR, **(B)** pre-contrast *T*_1_-weighted, **(C)** post-contrast *T*_1_-weighted (*T*_1_+C) images along with the corresponding **(D)** sRCBV map for a representative image slice. Though this is a non-contrast-agent enhancing tumor, this example illustrates that DSC rCBV imaging may also provide information in non-enhancing tumors.

More recently, a population-based digital reference object of brain tumor tissue was developed to evaluate the entire parameter space of tissue condition and imaging parameter settings (52, 53). The digital reference object results confirmed that the double-dose approach using a single-dose (0.1 mmol/kg) preload and single-dose DSC with Boxerman-Schmainda-Weisskoff correction was the most accurate and most robust method, being fairly insensitive to slight variations in parameter settings. Yet, another potential lower GBCA dose method emerged. By simply using a lower flip angle (30°) with TE = 30 ms (at 3 Tesla [T]) or TE = 54 ms (at 1.5 T), it was predicted that rCBV results comparable to the double-dose reference standard could be obtained. This was experimentally confirmed at 3 T in a multisite study of brain tumor patients (54). These results helped to inform a recent consensus recommendation specifically for DSC-MRI data acquisition in high-grade glioma, which includes both the double-dose reference standard and single-dose methods (55). Clinical MRI results together with rCBV maps, obtained using the double-dose reference standard from a patient with a grade 2 oligodendroglioma, are shown in **Figure 4**.

#### 3.2.3 Evidence from Clinical Studies

A systematic review and meta-analysis of studies between 2005 and 2015 included 17 DSC-MRI studies and concluded that individual studies, taken in an isolated form, showed encouraging results to differentiate tumor progression from treatment-related changes, with sensitivities and specificities in the 80%–90% range (56). Yet, widespread use has been hampered by the wide range of proposed rCBV thresholds reported (0.9–2.15). This variation is largely attributed to the statistically significant heterogeneity in how the DSC-MRI data are collected and analyzed (56). However, the studies reviewed preceded the recently published consensus recommendation that describes how best to acquire DSC-MRI data, and that a correction for leakage should be included. With widespread implementation of this consensus acquisition, it is hoped that greater consistency in mean rCBV thresholds will follow. A recent multisite study supports this contention (57). Several sites used their own post-processing platform to process the same DSC-MRI data whose acquisition was consistent with the subsequently published consensus recommendation regarding acquisition. A common threshold applicable to all sites could be determined to distinguish high- from low-grade tumor. This suggests that, with greater consistency in the acquisition of DSC-MRI data, agreement on thresholds to distinguish treatment-related effects from residual or recurrent tumor is also possible.

The practice of using the mean rCBV determined from the enhancing lesion ROI is another often-overlooked factor that may contribute to the wide range of reported rCBV thresholds. Rarely is a lesion composed of pure tumor or pure treatment effect. Instead, there is an admixture of tumor and treatment effect that will be unique for each patient and each cohort of patients. Yet, all studies described in the meta-analysis (56) used the mean rCBV from an ROI. Consequently, it is not surprising that each study reports a different threshold value because each study comprises a unique set of patients. Even if the acquisition and post-processing methods were equivalent, it is still likely that each study would report a different threshold to distinguish tumor from treatment effect.

**Figure 5:**
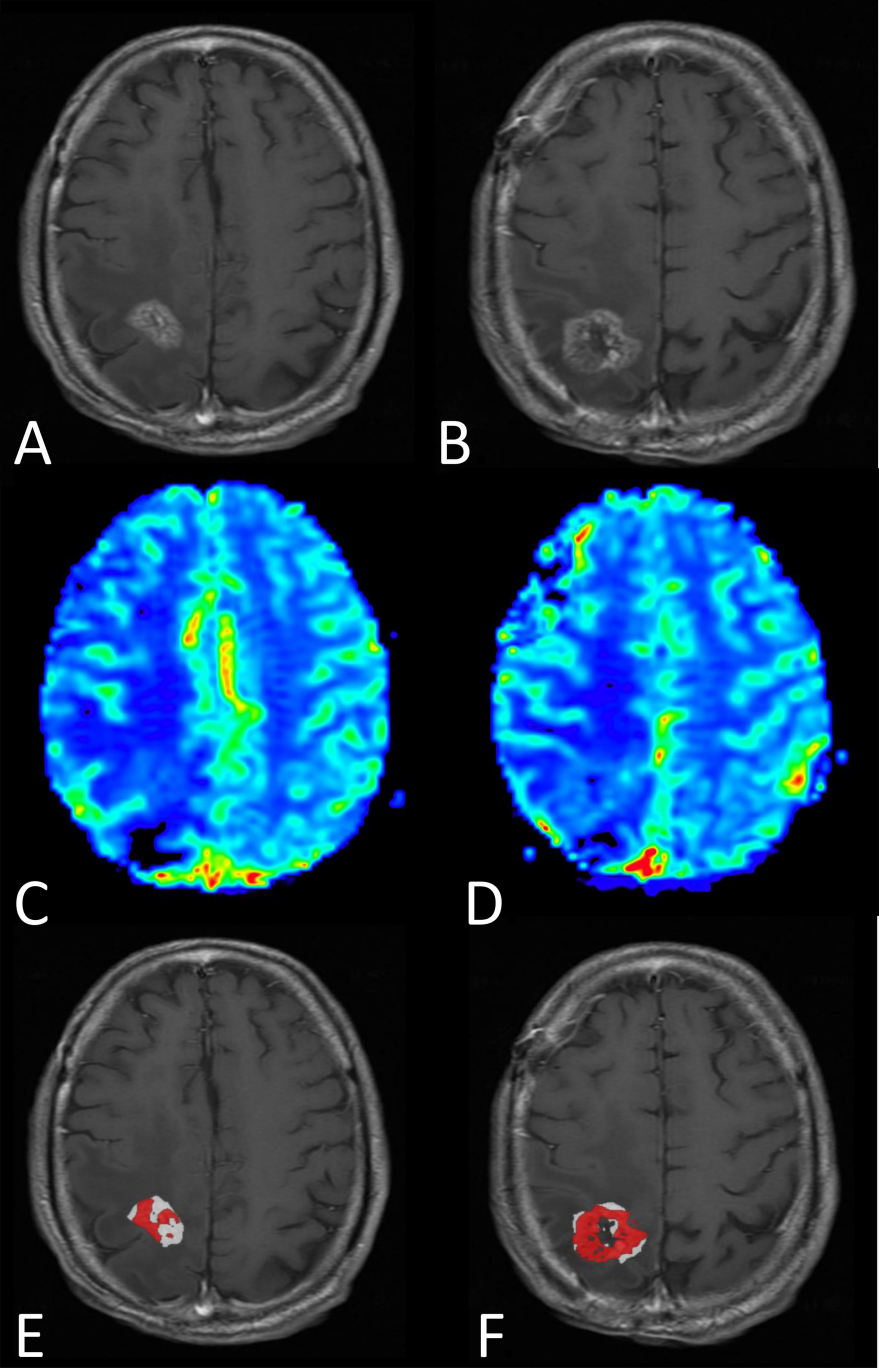
MRI results from a male patient diagnosed four months earlier with glioblastoma, IDH-wildtype, and treated with surgery followed by standard-of-care radiotherapy and concomittant temozolomide. Progression was questioned at the time of this MRI exam (three days prior to re-do surgery). **(A, B)** Post-contrast *T*_1_-weighted and **(C, D)** rCBV maps shown for two representative images slices. **(E, F)** FTB were created and show a mixture of tumor (red) and treatment effect (white), which is consistent with the pathologic diagnosis of tumor and treatment effect, respectively. Note that without the previously determined rCBV thresholds to distinguish tumor from treatment effect, the “blush” of higher rCBV noted on the rCBV maps would be difficult to interpret.

One proposed research solution to the determination of a widely accepted single threshold, is to use image-localized biopsy and spatially histopathologic correlation as described in two retrospective studies of high-grade brain tumors (58, 59). In these studies, only tissue confirmed to be either pure tumor or pure treatment effect were used to determine an rCBV threshold for distinction. In both studies, the consensus acquisition protocol was used, and the rCBV threshold was determined to be the same to distinguish high-grade tumor from treatment effect. A significant difference in tumor and treatment effect was also found for standardized rCBV (59). Biopsy-determined rCBV thresholds can be used to create voxelwise maps that distinguish tumor from non-tumor for the determination of the overall fraction of tumor burden within the enhancing lesion. These maps, referred to as fractional tumor burden (FTB) maps (60), provide a unique ability to visualize the admixture of tumor and post-treatment effects (**Figure 5**). The potential of FTB to predict response to treatment (61) and to distinguish between tumor or treatment effect, as confirmed by histopathologic examination of resected tissue samples (62), has been demonstrated. More recently, a prospective study (63) using image-localized biopsy tissue validated the ability of rCBV to predict tumor content (0–100%), demonstrating similar performance of nRCBV and sRCBV for the creation of FTB. Given that sRCBV does not require choosing a reference ROI, this represents an important step toward workflow optimization for the creation of FTB maps.

#### 3.2.4 Future Developments

Other DSC-MRI perfusion metrics have also demonstrated promise for providing clinically relevant information to evaluate treatment response. In a preclinical study, the distribution of mean transit times, determined from DSC-MRI, was shown to be dose-dependent and predictive of response to the anti-angiogenic agent Sugen11657 (64). In a related study, capillary transit time heterogeneities together with indices of estimated tumor oxygenation, both determined from the DSC-MRI data, demonstrated promise for predicting unfavorable therapeutic effects in patients with recurrent glioblastoma (65). Combined GRE and SE sequences, which can provide measures of mean vessel diameter, have been shown to correlate with brain tumor grade (45, 46) and response to anti-angiogenic treatment (64). Multi-echo perfusion sequences, such as SPICE (44) or SAGE (66), measure GRE and/or SE at multiple echo times (TEs), enabling implicit correction of *T*_1_ leakage effects and the data for determining both DSC-MRI and DCE-MRI perfusion parameters maps (67). Though the technology is still under development (68), these newer perfusion methods are likely to provide a wealth of information relevant to treatment monitoring in brain tumors. Furthermore, with the recent development of the brain tumor DSC-MRI digital reference object (52, 53), which can serve as a benchmark for all DSC-MRI methods (69), translation of these technologies for clinical use should take place more quickly than previously possible. The additional role to predict response to treatments is also emerging, as mentioned above, in the context of FTB.

#### 3.2.5 Summary, Strengths, and Weaknesses

Measures of rCBV derived from DSC-MRI have consistently demonstrated the ability to provide information about brain tumor growth and response to treatment that is not available with current structural MRI methods. Of all advanced techniques, DSC is the most extensively studied for clinically utility. With a power-injector available and a cannula placed for contrast agent administration, DSC-MRI data are easy to collect using commonly available imaging methods and requires only an extra 2–3 minutes of scanning. Despite the ease of use and valuable information provided, the widespread clinical use of DSC-MRI has been hampered by the need to correct for confounding leakage effects, which in turn has led to a variety of ways to collect and process this data. However, great strides have been made to overcome these limitations, with a recent publication clearly outlining how best to collect this data (Boxerman consensus) and a strong recommendation that leakage correction must be used in the post-processing. In this regard, the Boxerman-Schmainda-Weisskoff leakage correction (48) method has been the most commonly used and currently is the most widely recommended approach. Therefore, consensus regarding the collection and processing of DSC-MRI is being reached. Remaining challenges include the fact that the DSC-MRI acquisition is most often based on EPI. Although EPI provides the necessary temporal resolution, the spatial resolution is less good and significant signal dropout and geometric distortions can occur at air-tissue interfaces, such as near sinuses, with post-surgical blood products, or at resection sites. Therefore, efforts are underway to develop higher resolution fast imaging techniques with less sensitivity to these unwanted susceptibility effects. These efforts include the development of spiral-based sequences (44) or parallel imaging methods such as SENSE (70). With a complete solution composed of consistent acquisition and post-processing methods, all obtained with high temporal and spatial resolution, DSC-MRI should be widely accepted as a robust addition to standard brain tumor exam to assess treatment response.

### 3.3 DCE-MRI

#### 3.3.1 Methodology

**Figure 6:**
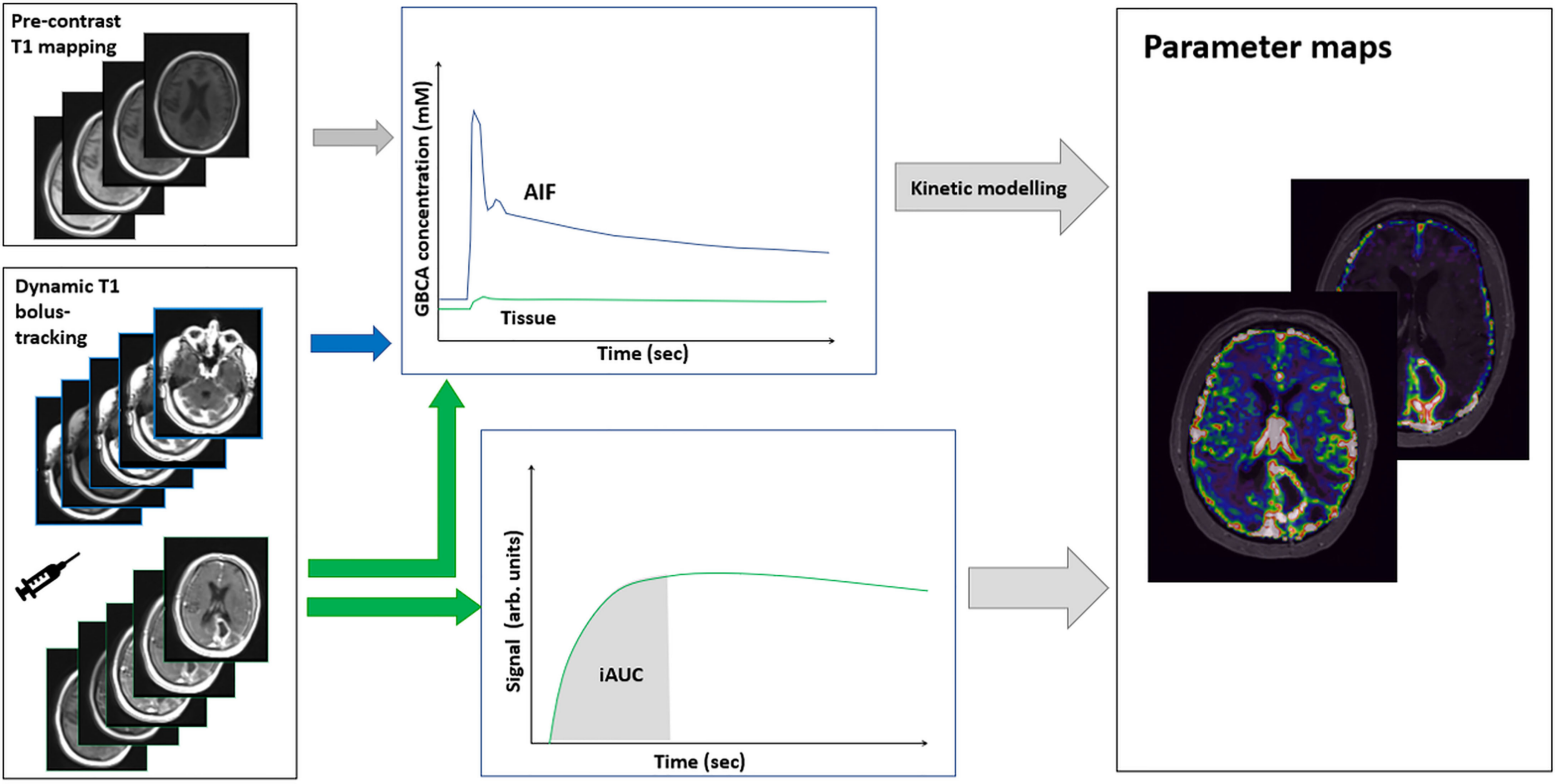
Acquisition and processing of DCE-MRI data. Using dynamic *T*_1_-weighted imaging, a signal increase is measured during bolus passage. From the preceding *T*_1_ mapping obtained by *T*_1_-weighted imaging at variable flip angles, the signal change can be converted into absolute concentrations of GBCA. From time concentration curves in arterial blood (AIF) and in tissue, absolute parameter maps can be produced by subsequent kinetic modeling. A simplified relative measure can be obtained from the area under the iAUC (initial part of the tissue signal curve), which reflects a mixture of blood flow, permeability, and blood volume.

DCE-MRI is based on dynamic *T*_1_-weighted imaging after intravenous administration of a GBCA (**Figure 6**). The contrast agent will increase the relaxation rate *R*_1_ = 1/*T*_1_, and the change in relation rate is linearly related to the concentration of the contrast agent *C* by


Equation 4 $$R_{1}=R_{1}^{0}+r_{1}C,$$

where $R_{1}^{0}$ is the relaxation rate before arrival of the contrast agent and *r*_1_ is the field strength specific relaxivity of the contrast agent. The exact relationship between signal (S) and change in relaxation rate depends on the sequence used. For the commonly used 3D *T*_1_-weighted spoiled gradient recalled echo sequence, with short TE << *T*_2_* so that *T*_2_* effects are negligible, the measured signal is related to the contrast agent by:


Equation 5 $$S(t)=M_{o}\sin(\alpha )\frac{1-e^{-\frac{TR}{T_{1}(t)}}}{1-\cos(\alpha )e^{-\frac{TR}{T_{1}(t)}}}=M_{o}\sin(\alpha )\frac{1-e^{-TR(R_{1}^{0}+r_{1}C(t))}}{1-\cos(\alpha )e^{-TR(R_{1}^{0}+r_{1}C(t))}},$$

where *α* is the flip angle and *M_o_* is the equilibrium magnetization. Only *T*_1_, and thus $R_{1}^{0}$ , are unknown and are usually estimated by preceding *T*_1_ imaging (typically obtained by performing *T*_1_-weighted imaging at variable flip angles).

**Figure 7:**
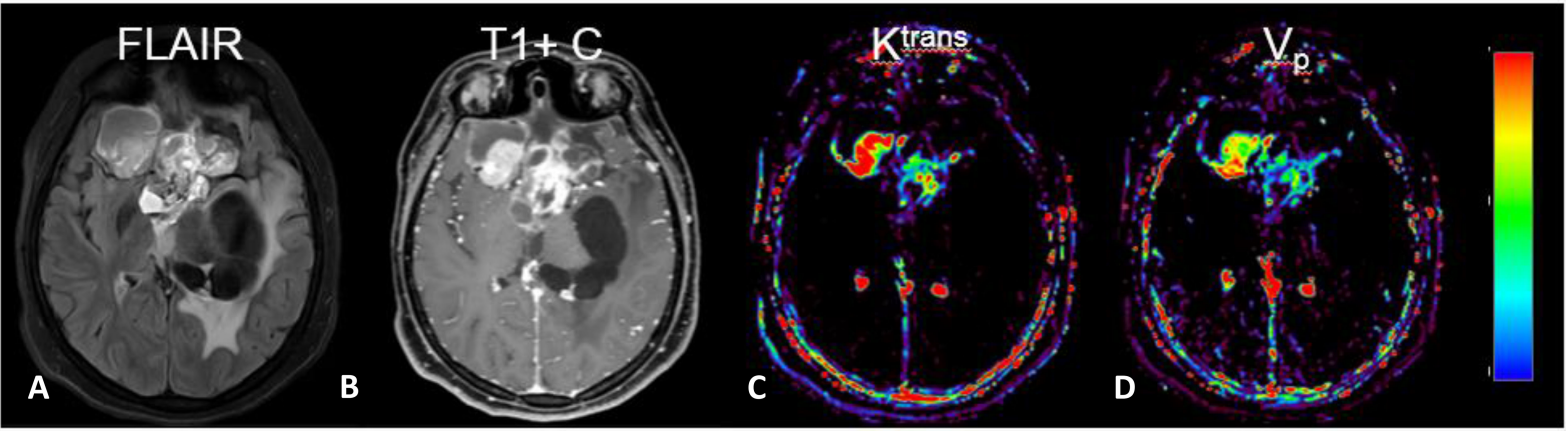
MRI with DCE parameter maps. **(A)** FLAIR and **(B)** post-contrast *T*_1_-weighted (*T*_1_+C) images of a patient with a primary brain tumor. In addition, the extended Tofts’ model was used to fit DCE-MRI data to generate parameter maps of **(C)** K^trans^ and **(D)** V_p_. (Images are courtesy of Imaging Biometrics LCC).

Various tracer kinetics models can be applied to the measured arterial and tissue concentration curves [see Ref. (71) for a comprehensive review]. The choice (and complexity) of the model, and thus the requirements for the imaging protocol, are dependent on the tissue and pathology of interest. For brain tumors, the most widely applied three-parameter two-compartment model (i.e., the extended Tofts’ model, **Figure 7**) provides voxelwise estimates of plasma volume fraction (V_p_), interstitial volume (V_e_), and K^trans^ (72), and thus information on permeability and blood volume, both indicators of neo-angiogenesis. For reliable estimation of K^trans^ from this model, a temporal resolution of < 10 sec (ideally ≤ 5 sec) and a total scan duration of 5 minutes following a single dose (0.1 mmol/kg) of GBCA are recommended in the proposed revised Quantitative Imaging Biomarkers Alliance (QIBA) profile (73). In the extended Tofts’ model, the parameter K^trans^ reflects both tissue perfusion (F) and PS. Except in extreme cases where permeability is very low (F >> PS and K^trans^ ≈ PS) or very high (PS >> F and K^trans^ ≈ F), the parameter K^trans^ is a mixture of F and PS. The errors/bias of the parameter estimates derived from the extended Tofts’ model may be substantial and depend on both the tissue (e.g., differences in low- compared to high-grade tumors) and the experimental conditions, e.g., temporal resolution (74). Still, this model may provide useful estimates of V_p_, K^trans^, and V_e_ also at low temporal resolution on 1.5 T systems.

Good repeatability of DCE using the extended Tofts’ model has been reported in normal tissue of both healthy subjects and patients with brain tumors (75), and moderate-to-good repeatability of K^trans^ similar to that of DSC nRCBV was found in contrast enhancing lesions of patients with glioblastoma during treatment (76). Nonetheless, a cross-vendor and multisite reproducibility study of quantitative *T*_1_-weighted imaging in a phantom found variable reproducibility dependent on both sequence and protocol used, field strength and range of *T*_1_ values (77). Furthermore, whilst studies of DCE reading and analysis reported good to excellent inter- and intraobserver agreement (78, 79), there was considerable influence from the software used (78).

#### 3.3.2 Future Developments

The current standard implementation of DCE using the extended Tofts’ model described in the recent QIBA guidelines (73) may be considered state-of-the-art for DCE brain tumor imaging, but it has changed little in recent years. Methods taking advantage of better signal-to-noise ratio (SNR) at 3 T allow higher temporal resolution, permitting quantification of F, e.g., by model free-deconvolution (80), and thus separation of F and K^trans^ by the four-parameter two-compartment exchange models (2CXM) (81, 82). Estimation of F is (among other factors) highly sensitive to errors related to undersampling of the input function; therefore, high temporal resolution (ideally 1–2 sec) is required and may be achieved by limiting coverage or spatial resolution. Simpler (model independent) approaches are based on initial area under the signal curve (iAUC), and reflect the initial accumulation of contrast by the tissue (irrespective of the mechanism). This approach alleviates the need for *T*_1_ mapping, measurement of arterial input function, and kinetic modeling, and it is possibly more robust across scanners and implementations (83, 84).

#### 3.3.3 Strengths and Weaknesses

DCE-MRI is the standard perfusion method outside the brain, e.g., prostate and breast, but it is less widely used than DSC-MRI in the brain. Nonetheless, DCE-MRI has several advantages compared with DSC-MRI for post-treatment imaging of brain tumors. First, DCE-MRI is a *T*_1_-weighted imaging technique and hence is less sensitive to the susceptibility artifacts in tissue adjacent to the skull base as well as within treated tumors that limit the coverage of *T*_2_*-weighted imaging in DSC-MRI (85). In fact, DCE-MRI might provide useful image quality in > 80% of uninterpretable DSC-MRI cases (86). Second, unlike *T*_2_*-weighted imaging, *T*_1_-weighted contrast leakage effects are not a cause of error when using DCE-MRI. Instead, they allow explicit modeling of permeability and can provide absolute parameter maps of tissue microvascular properties. Third, *T*_1_-weighted imaging in DCE allows easier and more accurate estimation of an AIF for kinetic modeling than EPI used for DSC-MRI. This and the conversion of blood and tissue signal to concentration of GBCA permit absolute quantification of physiological parameters, although quantitative parameter estimates probably are highly model specific. The main technical disadvantages of DCE-MRI compared with DSC-MRI are lower temporal resolution, need for *T*_1_ mapping, and increased scan duration. Clinically, there are no generally established cut-off points or diagnostic criteria using the standard implementation (the extended Tofts’ model). In the case of 2CXM analysis, a single small study investigated 2CXM DCE for pre-radiotherapy prediction of post-radiotherapy tumor progression (87), but no published clinical data regarding diagnostic accuracy are currently available, means of implementation are not standardized, and scanner and software solutions for advanced kinetic modeling are limited.

#### 3.3.4 Evidence From Clinical Studies

Compared with DSC-MRI, the available evidence from clinical studies is more limited. **Supplementary Table S2** shows studies of diagnostic accuracy for differentiating disease progression related and treatment-related effects. Two partially overlapping meta-analyses (56, 88) calculated pooled sensitivity and specificity of 88–89% and 85–86%, respectively, based in combination on a total of 10 studies published from 2011 to 2015 including 371 patients. It is noteworthy that the diagnostic accuracy was higher in studies with model independent analysis (88) than in studies with two- or three-parameter models, while most recent studies (and clinical practice) typically employ three-parameter models. Clinical multicenter brain tumor studies using DCE have been performed for tumor grading (79) and for evaluation of antiangiogenic treatment of recurrent glioma (76).

Several studies have made comparisons of the various DCE-MRI parameters with each other and with DSC-MRI. Optimal cut-off values determined by receiver operating curve (ROC) analysis and associated diagnostic accuracies from the individual studies are shown in **Supplementary Table S2**. Two large retrospective studies from the same group, each including more than 150 patients, found similar overall accuracy (area under receiver operating curve [AUROC]) of DSC-MRI and model free DCE-MRI for separation of progressive disease from pseudoprogression (89) and radiation necrosis (90), with DSC-MRI tending to provide higher specificity and lower sensitivity compared with DCE-MRI. Smaller retrospective studies comparing model-based analysis of DCE-MRI with DSC-MRI have reported variable findings, although single DCE-MRI parameters tend to provide lower diagnostic accuracy compared with DSC-MRI (**Supplementary Table S2**). The only prospective study comparing three-parameter analysis of DCE-MRI with DSC-MRI found AUROC of V_p_ and the model free analysis (iAUC) to be similar, and both slightly lower than DSC-CBV, whereas K^trans^ yielded the lowest AUROC (91). A prospective study including both high-grade glioma and metastases also found AUROC of V_p_ superior to K^trans^, and suggest improved accuracy when combing the V_p_ and K^trans^ (92). Other, mainly smaller, retrospective studies of three-parameter analysis have suggested K^trans^ or V_e_ outperform V_p_ (**Supplementary Table S2**), while a single study found no diagnostic value of any DCE-MRI parameter (93). The evidence does not converge toward recommending a specific parameter or cut-off point, but a visual analysis of parameter maps comparing tumor to normal tissues may be a clinically acceptable approach (94).

In summary, clinical studies have proven the potential of DCE-MRI for distinguishing treatment-related effects from recurrence, but the optimal DCE acquisition protocol, mode of analysis, and parameter of highest diagnostic value and optimal cut-off points remain to be established.

### 3.4 ASL

#### 3.4.1 Methodology

ASL is a perfusion imaging technique based on magnetically labeling water spins as they flow through the large brain-supplying arteries at the cervical spine level using radiofrequency (RF) pulses. An image of the brain is acquired after allowing for sufficient time for the labeled spins to reach the brain capillaries. The signal difference in magnetization between labeled images and non-labeled control images yields perfusion-weighted images using kinetic model estimations to calculate the quantitative CBF map (95). Typically, multiple label and control repetitions are acquired in alternating order, and their average subtraction value is used. A variety of ASL pulse sequences and associated analysis methods have been developed, which employ different strategies to control for unwanted labeling/subtraction effects and to overcome the intrinsically low SNR of the ASL signal. For a recent review focused on the ASL basic concepts and the current state-of-the-art acquisition and analysis approaches, see Ref. (35). New developments in ASL are also detailed by van Osch et al. (96).

##### 3.4.1.1 Labeling Schemes

**Figure 8:**
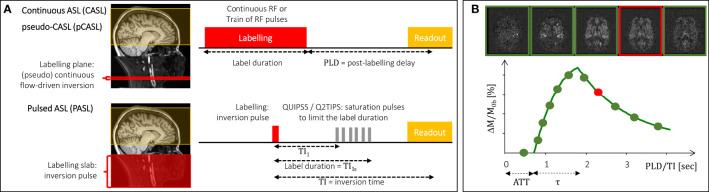
Basic principles of ASL perfusion imaging. **(A)** The two main labeling schemes (CASL/pCASL and PASL) are illustrated, in terms of the location and extent of the labeling region (left) and associated pulse sequences and their timings (right). **(B)** The magnetization difference between label and control images is illustrated by the difference images for five representative delays (top) and the underlying kinetic curve obtained based on the standard kinetic model (bottom). The ATT (arterial transit time) and label bolus duration (τ) are indicated. The red box and dot indicate a typical PLD/TI value for a single-delay acquisition.

The two main labeling schemes are continuous ASL (CASL) (97) and pulsed ASL (PASL) (98, 99), illustrated in **Figure 8**. While CASL relies on the flow-driven adiabatic inversion of arterial water spins as they flow through the labeling plane under the continuous application of a RF field, PASL is based on the instantaneous inversion of water spins within a wide slab upon the application of an adiabatic inversion RF pulse. A derivation of CASL was more recently developed, pseudo-continuous ASL (pCASL), which uses a train of RF pulses to induce flow-driven adiabatic inversion of arterial water spins (100). In this way, it retains the SNR advantage of CASL compared with PASL while allowing implementation in standard clinical MRI systems not possible with CASL. The advantages of pCASL relative to both CASL and PASL have made this the labeling scheme of choice for clinical applications (95). More advanced and purpose-specialized labeling schemes employ non-spatially selective labeling, which can be achieved by inverting spins based on their velocity, or acceleration, rather than their spatial position: velocity-selective ASL and acceleration-selective ASL (101).

##### 3.4.1.2 Image Acquisition

Fast image readout techniques should be employed, most commonly these are multi-slice 2D EPI but also segmented 3D methods such as 3D GRASE or 3D stack-of-spirals. While the latter are recommended for their higher SNR, the former is less sensitive to motion. Other acquisition parameters need to be selected, namely regarding background suppression and vascular crushing gradients, which may be used to minimize signal contributions from static and macrovascular spins, respectively. A critical choice is that of the time delay between labeling and image readout—post-labeling delay (PLD) in pCASL/CASL and inversion time (TI) in PASL (see **Figure 8A**). Most often, a single delay acquisition is performed: in this case, the delay should be long enough to ensure that the labeled spins have reached the tissues by the time signal is read out, so that it is not sensitive to variations in the arterial transit time or bolus arrival time, illustrated in **Figure 8**. Additionally, in PASL, saturation pulses should be applied in order to control the label duration, using QUIPSS II (102) or Q2TIPS (103) approaches (see **Figure 8A**). Because the label, and hence the perfusion signal, decay with longitudinal relaxation (*T*_1_ of arterial blood up to the capillaries and *T*_1_ of tissue after exchange), the trade-off between insensitivity to arterial transit time and sufficient SNR must be considered when choosing the value of PLD/TI. Alternatively, multiple delays may be sampled and an appropriate kinetic model fitted to the data to estimate CBF as well as arterial transit time. For multi-delay acquisitions, optimal sampling schemes (104, 105) or more efficient sampling schemes such as time-encoded pCASL (106), should be considered.

##### 3.4.1.3 Perfusion Quantification

For perfusion quantification, tracer kinetic models have been developed with different levels of complexity. A standard kinetic model of ASL signals was originally developed by Buxton assuming a single well-mixed compartment and instantaneous exchange of water between blood and tissue (107) (see **Figure 8B**). Various extensions of this model have since been proposed, namely to account for restricted water exchange/vessel permeability using two compartment models (108, 109), macrovascular (as well as microvascular) contributions (110), partial volume effects (111), or flow dispersion (112). Simplifications of the standard model have also been proposed (95) to obtain CBF measurements based on single-delay ASL acquisitions:


Equation 6 $$\text{In pCASL}:CBF=\frac{\lambda \Delta Me^{\frac{PLD}{T_{1b}}}}{2\alpha T_{1b}M_{0b}(1-e^{-\frac{\tau }{T_{1b}}})}$$

Equation 7 $$\text{In PASL}:CBF=\frac{\lambda \Delta Me^{\frac{TI}{T_{1b}}}}{2\alpha TI_{1}M_{0b}}$$

where ΔM is the control-label magnetization difference, M_0b_ is the equilibrium magnetization of arterial blood, *T*_1b_ [s] is the longitudinal relaxation time constant of arterial blood, λ is the blood/tissue partition coefficient for water, τ [s] is the label bolus duration, and α is the labeling efficiency. In order to quantify CBF in absolute units, calibration of ASL measurements must be performed. This involves normalizing the control-label difference images, ΔM, by the equilibrium magnetization of arterial blood, M_0b_ (113). Since M_0b_ cannot be measured directly *in vivo*, different methods have been proposed to estimate it based on the measurement of the tissue M_0_, typically using a proton-density image acquired with the same readout as the ASL images but with a long repetition time (TR).

##### 3.4.1.4 Consensus Recommendations

In an attempt to harmonize ASL perfusion measurements across studies, a consensus paper was published in 2015 with recommendations regarding the most appropriate implementation for clinical applications (95). In particular, the combination of pCASL with a segmented 3D readout was recommended for its superior SNR; however, PASL and 2D readouts still are often used. The use of background suppression without vascular crusher gradients is also recommended. Although ASL may be performed at 1.5 T, the recommended field strength is 3 T. In this paper, a calibration procedure and a simplified kinetic model were also proposed for CBF quantification based on single delay measurements. Although the consensus paper resulted in part from the efforts of a European network focused on dementia, the recommendations were made for clinical applications more generally. In fact, this consensus paper resulted in the implementation of a recommended sequence by MRI vendors, and this has indeed been adopted by an increasing number of studies. However, the consensus paper did not take tumor studies into key account, nor does it discuss peculiarities of tumor ASL imaging, which may influence the optimal application of ASL in tumor imaging under the recommended conditions. Several studies have assessed the test-retest repeatability of ASL CBF measurements, including the recommended single-delay pCASL implementation (114, 115) but also more complex protocols (116, 117). A reproducibility study has been performed for ASL CBF measurements obtained with commercial implementations of ASL across from major MRI vendors (118). This, however, preceded the consensus paper and results may be improved now that vendors have generally adopted these recommendations. Regarding multisite reproducibility, only one study has partially investigated this by assessing CBF in specific brain regions (115). All of these reliability studies have been performed on healthy subjects, and no studies in patients with brain tumors have been reported.

#### 3.4.2 Strengths and Weaknesses

**Figure 9:**
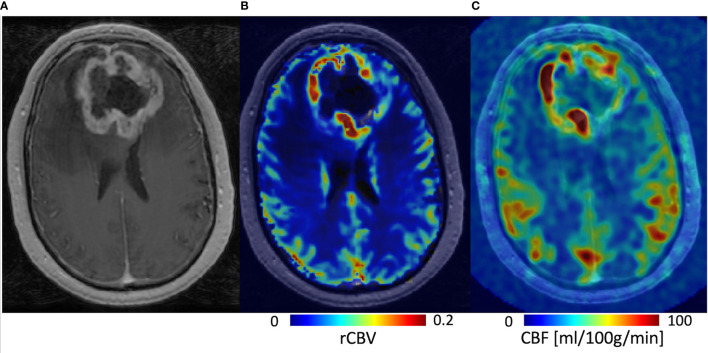
Three-months post-radiotherapy follow-up in a 58-year-old patient diagnosed with a glioblastoma, IDH-wildtype, studied on a 3T GE MRI system. **(A)** The post-GBCA *T*_1_-weighted series shows thick rim enhancement, which could theoretically also be pseudoprogression after recent completion of standard-of-care radiotherapy and concomittant temozolomide. **(B)** The DSC rCBV map, acquired with 2D EPI (TE/TR = 45/1500 ms), indicates general hyperperfusion of the enhancing outer tumor rim, which makes early true progression more likely and is thus decisive for further therapy planning. **(C)** The ASL CBF map, which was acquired with 3D pCASL using white paper settings, shows hyperperfusion concordant with the DSC rCBV map but without the need of gadolinium administration.

In contrast to DSC-MRI and DCE-MRI, ASL techniques do not require the injection of a contrast agent. As a consequence, fast repeated measurements on individual patients can be performed. Moreover, the absence of potential gadolinium deposition effects also allows ASL to be more widely used in pediatric patients and in patients with impaired renal function. Relative to DSC-MRI, ASL has the additional advantage that perfusion measurements are not tampered by BBB permeability effects. Even after leakage effects have been corrected for, DSC perfusion imaging is based on GBCA as an intravascular tracer, which limits its ability to yield true perfusion measurements. In contrast, ASL is based on water as a diffusible tracer, allowing it to truly measure perfusion at the capillary level (**Figure 9**). Additionally, susceptibility effects leading to signal dropout and geometric distortions are less prominent since shorter echo times are used.

Despite the potential advantages relative to DSC-MRI and DCE-MRI, the main weakness of ASL remains its intrinsically low SNR. This has led to the proliferation of methodological options and limited accessibility in clinical sites, which have hindered its wider application so far. Besides the general lack of robustness that results from the low SNR, ASL is also more sensitive to motion artifacts than DSC-MRI or DCE-MRI, due to the inherent image subtraction as well as the frequent use of 3D readout. Additionally, single-delay ASL acquisitions may have macrovascular artifacts or underestimation of tissue perfusion in areas with prolonged arterial transit times, e.g., in patients with macrovascular stenosis.

#### 3.4.3 Evidence From Clinical Studies

An overview of clinical studies published is provided in **Supplementary Table S3**. All studies employed commercial pCASL sequences with a single delay. Three of the listed studies were part of a recent meta-analysis of a total of 160 patients, which reported a pooled sensitivity and specificity of 79% and 78%, respectively to differentiate true progression from post-treatment related effects by ASL (119). All other studies also reported reported fair to good diagnostic accuracies for differentiating true progression from post-treatment related effects. The largest retrospective study included 69 patients (both low and high-grade gliomas) and reported sensitivity and specificity of 74% and 82%, respectively, for separation of progression from post-treatment related effects (120). In the two largest prospective studies, one study found both high sensitivity (94%) and specificity (92%) for separation of post-treatment related effects from true progression among 42 patients with treated high-grade gliomas (121), while the other reported low sensitivity (65%) but very high specificity (100%) among 58 patients (both low- and high-grade gliomas) in the context of pseudoprogression (122).

#### 3.4.4 Future Developments

Studies exploring the utility of ASL for the identification of post-treatment related effects in high-grade glioma are limited to basic implementations of the technique and have technical and clinical variabilities as well as small sample sizes. Despite this, ASL continues to undergo substantial advances yet to be fully exploited in this context (96). In particular, advanced ASL techniques based on multiple-delay ASL measurements combined with appropriate kinetic models may be employed to obtain measurements of perfusion-related parameters besides rCBF (35). Of special interest in glioma is the putative possibility to obtain vessel permeability measurements by using multi-compartment kinetic models incorporating water exchange between blood and tissue (108, 109). For now, the biggest challenge is less the technical development and more the harmonization of current protocols, which would lead to clearer comparison between studies with the potential downstream effect of generating a higher level of evidence.

### 3.5 An Overview of Diffusion Techniques

Various diffusion MRI techniques can be applied to glioma treatment response assessment including standard diffusion-weighted imaging (DWI), apparent diffusion coefficient (ADC) analysis, diffusion tensor imaging (DTI), diffusion kurtosis imaging (DKI), intravoxel incoherent motion (IVIM), as well as a multitude of microstructural modeling approaches, such as neurite orientation dispersion and density imaging (NODDI) (123) or restricted spectrum imaging (RSI) (124); free water imaging (FWI) (125); and vascular, extracellular and restricted diffusion for cytometry in tumors (VERDICT) (126).

The basic DWI sequence achieves diffusion contrast based on different gradient pulses in between the excitation and refocusing pulses of a basic SE experiment (127). This basic sequence produces images that highlight diffusional water molecule motion by signal loss. It has been extended to capture information of diffusion in different directions (128) and over different time periods. The gradient strength and duration are expressed in different b-values with a unit of s/mm^2^. Typical b-values for brain imaging are up to a single (also called single-shell) b-value of 1000 s/mm^2^ and the signal decay up to this b-value can be approximated by a Gaussian distribution. Notably, lower (50–250 s/mm^2^) b-values are associated with signal from microvascular perfusion rather than Brownian diffusion motion, which is important in, for example, IVIM (129), a method that aims to recover the pseudo-diffusion coefficient of blood using several b-values (also called multi-shell) in its modeling approach. Note that multi-shell acquisitions may also be used in other modeling approaches. Higher b-values of > 1000 s/mm^2^ are associated with more restricted and hindered environments employed in non-Gaussian (such as DKI) and microstructural models.

**Figure 10:**
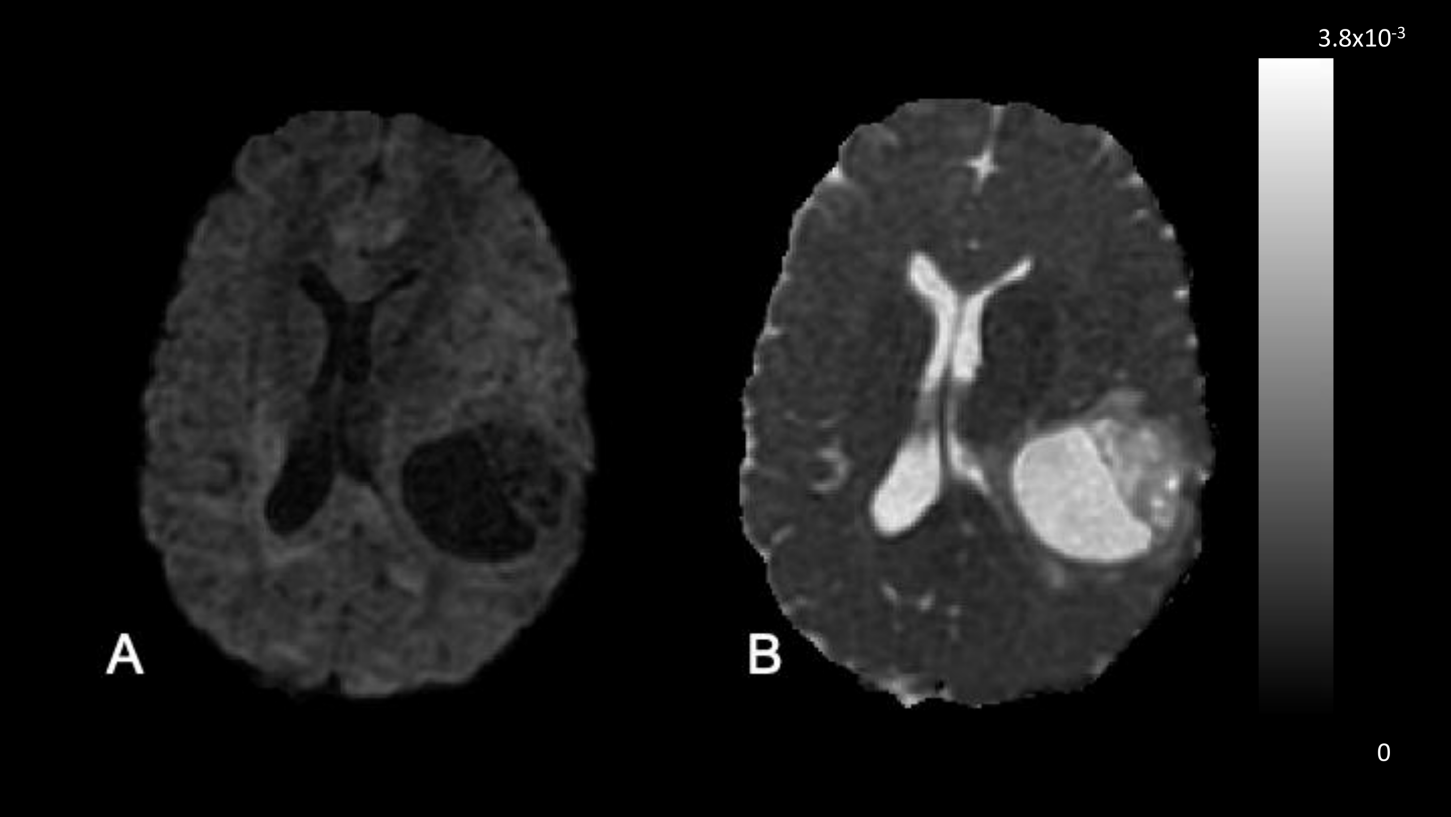
DWI and ADC. These images were acquired in a 50-year-old treatment-naive female patient with glioblastoma, IDH-wildtype. **(A)** DWI (b = 1000 sec/mm^2^). The necrotic cavity and necrotic rim are DWI hypointense, due to the diffusing spins having moved between the time of the excitation and the refocusing pulse. **(B)** ADC map. In the ADC map fast diffusion is highlighted by hyperintensity. The combined interpretation of both images gives the qualitative inference that this more likely represents a cavitating or cystic-like tumor with low cellularity rather than a brain abscess or lymphoma.

The ADC is the best-known diffusion parameter. By scaling the diffusion image with a non-weighted image, quantitative maps can be produced. This quantitative technique is available on any commercial MRI machine, which makes the integration in clinical trials attractive. Qualitative visual interpretation is also possible and gives an impression of cellular density and water content and can help in clinical differential diagnostics (**Figure 10**).

Extending the diffusion methodology to sensitize the signal over different directions, the DTI approach has seen widespread application due to its methodological and mathematical simplicity and elegance (130). DKI models (131) focus on the deviation from the Gaussian signal. This model is more sensitive to heterogeneity and complex tissue architecture, rendering parameters that are correlated with biological tissue properties and reflecting the organization of tissue microstructure (132). This approach may deliver biomarkers more relevant to disease interpretation, such as in heterogeneous environments, and dense architecture, such as solid tumors (133). A different approach to overcome the limitation of the Gaussian model is by explicitly modeling it. The FWI model can be used both to visualize the Gaussian signal (free water) and to remove it (free water corrected) (125) using a multi-shell acquisition. This may be useful to distinguish a large area of edema or necrosis from residual tumor and can be used to make DTI fiber tracking in the presence of such processes more accurate (134). Other multi-shell models aim to advance the understanding of neuronal diffusion parameters, such as NODDI (123) or RSI (124). Although NODDI has been designed entirely for use in normal brain tissue, the RSI model has been adapted for use in tumors (135).

### 3.6 DWI

#### 3.6.1 Methodology

The b-value dependent diffusion signal in a classical diffusion acquisition is calculated based on the Stejskal-Tanner equation (127), where S_b_ is the signal depending on the apparent diffusion coefficient ADC and the diffusion gradient length δ, separation Δ, and strength G. The b-value is defined as $b=\gamma^{2}\cdot G^{2}\cdot \delta^{2}\left(\Delta -\frac{\delta }{3}\right),$ where γ is the gyromagnetic ratio. Assuming a pure Gaussian diffusion of water and a log-linear signal decay with increasing b-value:


Equation 8 $$S_{b}=S_{0}e^{-b\cdot ADC}$$

By acquiring a diffusion weighted image S_b_ and an unweighted image S_0_, the apparent diffusion coefficient ADC can be calculated as:


Equation 9 $$ADC=\log\frac{S_{b}}{S_{0}}\cdot -\frac{1}{b}$$

If more than one DWI S_b_ exists, ADC may also be derived by calculating a least squares fit to the Stejskal-Tanner equation.

To sensitize the diffusion image S_b,_ the diffusion gradient pulse may be played out on each of the three gradient axes individually, or on all three gradients at the same time (so-called tetrahedral scheme) (136). If played out on each axis individually, the images need to be averaged before the ADC map is calculated. Therefore, to calculate an ADC image, four separate volumes have to be acquired: the unweighted image S_0_ and three S_b_ images for each of the individual gradient axes. To reduce the time it takes to acquire the sets of images, EPI (137) is commonly performed. EPI uses blipped line encoding to fill out k-space more quickly. Although this technique is incredibly fast, as with perfusion EPI acquisition, it is sensitive to susceptibility changes in the local magnetic field, often leading to artifacts near large tissue-air boundaries e.g., close to the frontal sinuses of the skull.

#### 3.6.2 Strengths and Weaknesses

Diffusion-weighted sequences usually have a fast acquisition time, which makes them suitable for anxious or poorly compliant patients, both of which are common in the brain tumor patient population. DWI sequences are globally ubiquitous and well-standardized across centers with good dissemination of clinical knowledge of image interpretation compared to more advanced methods. They allow the discrimination of differential diagnoses such as stroke mimicking tumor progression or infection in the surgical cavity. On the other hand, DWI is affected by geometric distortions close to bone and air, which can affect diagnostic quality in adjacent brain structures. Similar artifacts can occur in the resected surgical cavity due to blood products.

#### 3.6.3 Evidence From Clinical Studies

**Figure 11:**
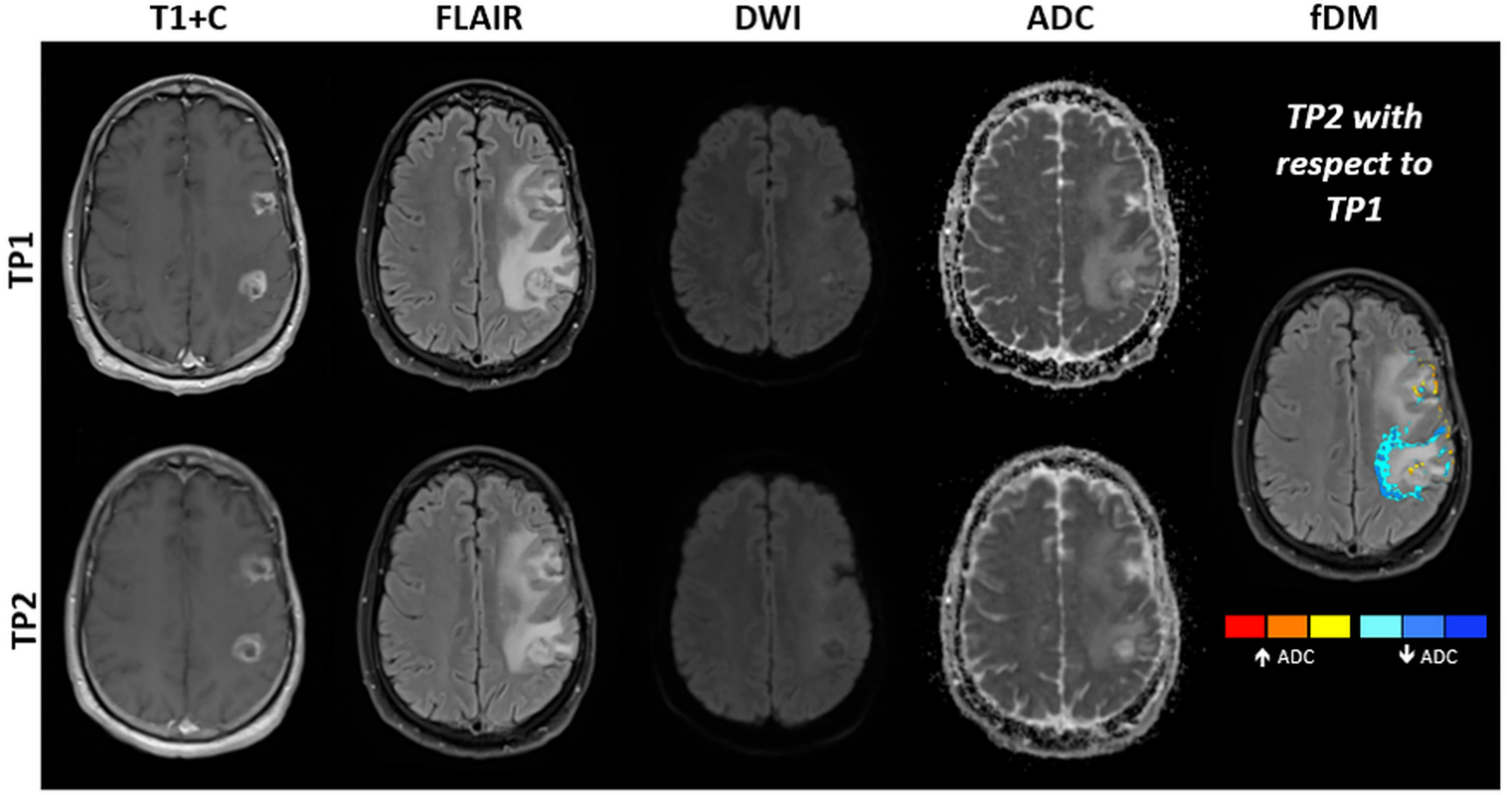
MRI obtained in a 48-year-old patient with mulitfocal glioblastoma, IDH-wildtype. Shown are post-contrast *T*_1_-weighted images, FLAIR images, DWI at b = 1000 s/mm^2^ and ADC maps obtained at approximately two (TP1) and three months (TP2) after standard-of-care radiotherapy and concomittant temozolomide. Subtraction of ADC obtained at TP1 from ADC obtained at TP2 results in the creation of fDMs, which show where ADC is increasing (red to yellow) or decreasing (blue). Decreasing ADC (blue regions) is suggestive of increasing tumor cell density. Surgical resection performed one week after TP2 revealed an admixture of tumor recurrence and post-treatment related effects. Note that interpretation can be complicated by resolving edema between time points, which may also show decreased ADC.

Progressive disease in high-grade gliomas is characterized by tumor hypercellularity manifesting as low ADC values (138–141), which confer poor survival (142–144). In contrast, an increase in ADC may indicate response to chemoradiotherapy (138, 145, 146) (**Figure 11**). Similar findings have been seen in other brain tumors, in tumors outside the central nervous system, and after high-grade glioma immunotherapy (138, 147–149). It is noteworthy that high ADC values have also been associated with the peritumoral *T*_2_ hyperintensity of glioblastoma, albeit inconsistently. A variety of advanced analyses using histogram-based techniques, either at a fixed time or longitudinally, capture tumor heterogeneity and can be applied to ADC maps to characterize therapy (138, 150, 151). One study showed that the fifth percentile on a cumulative histogram of ADC, obtained at a b-value of 3,000 sec/mm^2^, was lower in progressive disease than in pseudoprogression (P < 0.001) (151). In two single-site studies, a clear cut-off of a mean ADC proved to be successful in predicting pseudoprogression (152, 153) with ADC ≤ 1300 × 10^−6^ mm^2^/s (sensitivity 100%, specificity 100%) and ≤ 1313 × 10^− 6^ mm^2^/s (sensitivity 98.3%, specificity 100.0%), respectively for true progression.

A recent meta-analysis, which contained six eligible studies with a total of 214 patients, concluded that quantitative ADC is an effective approach for differentiation of glioma recurrence from pseudoprogression, and can be used as an auxiliary tool to diagnose glioma progression: sensitivity was 0.95 (95% confidence interval [CI] = 0.89 – 0.98), specificity was 0.83 (95% CI = 0.72 – 0.91) (154). Another meta-analysis showed that quantitative ADC has moderate diagnostic performance in differentiating glioma recurrence from radiation necrosis (sensitivity: 0.82 (95% CI: 0.75 - 0.87); specificity: 0.84 (95% CI: 0.76 - 0.91). The authors recommended not using diffusion MRI alone in differentiating between glioma recurrence and radiation necrosis (155). The latter analysis was supported by a meta-analysis comparing advanced imaging techniques that gave sensitivity and specificity results of 0.71 (95% CI: 0.60 - 0.80) and 0.84 (95% CI: 0.77 - 0.93), respectively, providing the lowest diagnostic accuracy among advanced MRI techniques analyzed (also MRS, DSC-MRI and DCE-MRI) (156). Ordinary ADC maps were found to be inferior in spatially discriminating treatment response compared with DSC-derived perfusion measures (59). Another study could not find any difference between perfusion and diffusion metrics in the ability to discriminate between recurrent tumor and pseudoprogression (157). In a different study, neither DSC-MRI nor DWI were found to be predictive of tumor recurrence (158).

Incorporating diffusion metrics into radiomics models improved diagnostic performance for identifying pseudoprogression over a model with only structural imaging volumes (89). A retrospective study employing a dictionary-learning approach was able to train its model to identify pseudoprogression from DTI data (159). The performance accuracy of radiomics and machine learning approaches in MRI are described in detail in Part 2.

#### 3.6.4 Future Developments

A few studies have used longitudinal ADC maps, namely parametric response maps (PRMs) (160) or functional diffusion maps (fDMs, **Figure 11**) (145), to discriminate progression from pseudoprogression (161–163). Whilst these promising methods have been at the research stage for more than a decade, more evidence is required to translate these as routine clinical tools.

Since the initial development of fDMs (145, 164, 165), many other studies have assessed the potential of fDMs in treatment response evaluation in glioma (164, 166, 167). A study of 50 patients with glioma proved that the rate of change in fDM “hypercellular” volumes within hyperintense fluid-attenuated inversion recovery (FLAIR) regions of interest, predicted tumor progression, time-to-progression, and overall survival for both cytotoxic and antiangiogenic treatments earlier than standard anatomical imaging (167). Another study including 20 brain tumor patients found that fDMs predicted patient treatment response at three weeks from the start of chemoradiotherapy, revealing that early changes in tumor diffusion values could be used as a prognostic indicator of subsequent treatment response (146). The preliminary conclusion regarding fDMs is that this imaging biomarker has the potential to track treatment response in brain tumor patients in both enhancing and non-enhancing tumors. A recent study employing ADC histogram analysis, found that low-ADC tumor volume shrinkage has the same predictive power as fDMs (168).

A small study assessing the use of PRMs with "relative" ADC, where the relative ADC represents a ratio between tumor and contralateral normal-appearing tissue, showed the potential in differentiating true progression from pseudoprogression (169). Voxel-based PRMs of ADC were used to predict early tumor progression (170). Compared with traditional response criteria (Macdonald or RANO criteria (171)), a PRM has the potential to allow individualization of treatment seven to eight weeks earlier than the traditional response criteria (172), since PRM stratification can be determined during the radiotherapy and concomitant temozolomide treatment phase.

Given that DWI is acquired in most routine MRI examinations, post-processing methodologies leveraging multiparametric combinations, also described in Part 2, have often incorporated diffusion techniques. This may be with or without the application of machine learning approaches. For example, a machine learning model that used FLAIR and DWI images was able to predict tumor recurrence with higher accuracy than using either modality by itself (173). Likewise, analysis with a multiparametric clustering approach was shown to be superior to a single-modality approach (174, 175).

In summary, study designs will likely improve as large prospective studies are performed using published study protocols and this will provide a higher level of evidence for their translation as routine monitoring biomarkers for high-grade gliomas (176). Analyses are also likely to become more complex using histogram analyses or machine learning approaches, which can integrate longitudinal and multiparametric data. The beginnings of this development can already be seen (177).

### 3.7 IVIM and DKI

#### 3.7.1 Methodology

IVIM and DKI are based on standard diffusion acquisition techniques. Standard DWI with ADC maps as a reference assume that water diffusion in tissues is Gaussian and that the diffusion signal will fall log-linearly with increasing b-value. However, the reality is different due to cell membrane interactions in anisotropic nerve fibers (and glial cells and their products such as myelin) and intravascular water in capillaries. Both IVIM and DKI try to assess the non-Gaussian and perfusion components in the diffusion signal, IVIM at the low end of the b-value axis of the signal-decay curve, assessing the perfusion-dependent component, and DKI on the high end of the b-value axis, above 1000 mm^2^/s.

The physiological premise behind IVIM is that due to the complex networks of capillaries, the flowing water-bound protons appear to move randomly, thereby simulating a Gaussian diffusion process. This pseudo-diffusion component can be expressed through various extensions of the basic Stejskal-Tanner equation, of which the biexponential model is possibly the most frequently applied in IVIM (178):


Equation 10 $$\frac{S(b)}{S(0)}=f\cdot e^{-b.(D^{*}+D_{blood})}+(1-f)\cdot e^{-b\cdot D_{tissue}}$$

with f being the capillary blood perfusion fraction in the diffusion signal, D* the pseudo-diffusion coefficient in the capillaries, and D true diffusion in either blood or tissue.

However, model fitting can become more stable, and thus reliable for application, if the model is simplified by separating diffusion into the fast pseudo-diffusion component of flowing water molecules, D*, and the slow component of water molecules moving by thermal diffusion, D, without further subdivision of tissue and blood components:


Equation 11 $$\frac{S(b)}{S(0)}=f\cdot e^{-b\cdot D^{*}}+(1-f)\cdot e^{-b\cdot D}$$

At high b-values, cell membranes become an influential factor on the diffusion signal. Therefore, the kurtosis effect, or the width and amplitude deformation of the Gaussian diffusion probability curve, needs to be incorporated as a dimensionless kurtosis factor K (131):


Equation 12 $$\frac{S(b)}{S(0)}=f\cdot e^{-bD^{*}}+(1-D+f)\cdot e^{-bD+\frac{1}{6}b^{2}D^{2}K}$$

The choice of b-values influences the shape of the fitting curve and therefore the parameter results. For IVIM, this means that more b-values should be chosen in the spectrum below 250 mm^2^/s. Currently, there is no consensus on how many or exactly which b-values should be chosen for IVIM and DKI measurements.

#### 3.7.2 Strengths and Weaknesses

IVIM and DKI are both relevant techniques in neuro-oncological imaging because they can potentially assess important tumor tissue properties such as microperfusion and cellularity. They are also non-invasive. Multiple b-value acquisitions, however, can extend scan time. Choosing fewer b-values will shorten scan time but potentially will reduce the accuracy of the signal curve fit. Furthermore, IVIM and DKI, like all diffusion techniques, are heavily influenced by scanner-dependent field homogeneity as well as magnetic distortion caused by the tissue examined, which can limit their application e.g. in tissues close to air-filled spaces. There are many other limitations in the models used for post-processing, which can either underestimate or overestimate IVIM and DKI parameters. A detailed elaboration on the issues of fitting and parameter calculation for both IVIM and DKI was published in 2017 (131). The influence of model parameter selection to calculate the perfusion fraction in IVIM, for example, has been shown in gliomas (179).

#### 3.7.3 Evidence From Clinical Studies

The ADC and perfusion fraction (f) extracted using a previously validated simplified IVIM model (150, 180) were used to assess the prediction of early progression in IDH-wildtype glioblastoma (181). Higher ADC_T2-FLAIR_ at baseline, lower f_T2-FLAIR_ at the 10th (of 30) radiation fraction, and lack of increase in ADC_T2-FLAIR_ at the 20th radiation fraction compared with baseline, were associated with early progression. IVIM metrics were not associated with overall survival, while higher f_T2-FLAIR_ at the 10^th^ radiation fraction was associated with longer progression-free survival (181). In a study of 51 patients (182), a histogram analysis of IVIM metrics was able to differentiate true progression from pseudoprogression, with ROC analyses identifying the 90^th^ percentile for perfusion to be discriminant with a sensitivity of 87.1%, and a specificity of 95.0%. A comparison study of different ADC modeling approaches found that all computed metrics, including those using the IVIM model, can differentiate tumor recurrence from pseudoprogression (100).

In a recent study of 32 glioblastomas, DKI showed high diagnostic accuracy, up to 88%, for differentiating between tumor progression and post-treatment related effects (183). In a comparison with DTI, DKI was found to be superior in predicting recurrence (184). DKI recently was applied to investigate treatment response and pseudoprogression in several studies (184–186) and these found that DKI was superior in assessing treatment response when compared to ADC and DTI. However, one of the studies found only limited benefit when compared to standard ADC measurements (185).

#### 3.7.4 Future Developments

It is potentially possible to render a tumor angiogram and elastographic information based on IVIM and DKI measurements (178). Because vascular and elastographic tumor imaging are upcoming techniques in glioma imaging (187, 188), IVIM and DKI may be applied to such advanced applications. A more important development in the near future will be determning the optimal choice of the number of b values needed for a reliable fit for IVIM and DKI (189).

### 3.8 DTI

#### 3.8.1 Methodology

By sampling the diffusion signal with at least six different directions, the information can be grouped into a so-called tensor, a multi-dimensional mathematical construct that adheres to mathematical operators (139) and can be used to determine diffusion magnitude (mean diffusivity) and degrees of diffusion anisotropy (fractional anisotropy [FA]). The estimated directionality can be visualized through post processing techniques using ellipsoids or fibers. DTI obtained by acquiring repeated DWI in different non-collinear directions can lead to some quantitative parameters, such as FA, mean diffusivity, axial diffusivity, and radial diffusivity. FA is an index between 0 and 1 to assess the degree of diffusion asymmetry in a voxel in terms of its eigenvalues. Because anatomical tracts are anisotropic, FA maps show where tracts are passing, or changing in disease, e.g., through destruction of these due to tumor cell invasion or sometimes treatment. A limitation of the diffusion tensor model is the assumption that the diffusion of water will always be Gaussian (190). This is true for diffusion within a singular environment (i.e., without different compartments), but due to imaging voxels being relatively large, it rarely points to a complete picture in human tissue.

#### 3.8.2 Strengths and Weaknesses

All scanner manufacturers have protocols to acquire DTI data and calculate mean diffusivity and FA maps as part of a post-processing step on the console. However, there is no common framework and a lack of consensus regarding how many directions or which b-values shells should be chosen. As in DWI, EPI-related artifacts such as geometric distortions and signal loss present challenges in superimposing DTI-derived maps on structural imaging. Although tractography is currently less relevant as a monitoring biomarker and largely beyond the scope of this position statement, it is noted that some scanner manufacturers offer the ability to perform tractography. These algorithms are often a “black-box,” making comparison and reproducibility difficult. Commercial and non-commercial software packages exist, employing both deterministic and probabilistic algorithms. It is noteworthy that these require considerable training and present challenges when integrating the process with clinical workflows.

#### 3.8.3 Evidence From Clinical Studies

After standard-of-care radiotherapy and concomittant temozolomide, when compared with pseudoprogression, the FA in contrast-enhancing tumor was higher in true tumor progression (162, 191, 192) and even appears to increase when compared to previous measurements (163). Whilst higher FA in progression has been postulated to occur due to the orientation of overproduced extracellular matrix in glioblastoma, other authors reported no difference in FA when pseudoprogression and progression were compared (141, 193). DTI-based maps of tissue isotropy and anisotropy have also been successfully used to predict recurrence of glioblastoma (194).

#### 3.8.4 Future Developments

Clinical studies aiming to optimize the multiple b-value shells and number of directions selected, are required before consensus standardization will support translation into the clinic routinely. This is being enabled by the dissemination of sequences and greater accessibility of post-processing pipelines without the need for advanced programming skills.

### 3.9 Other Advanced Diffusion Techniques

#### 3.9.1 Methodology

Multiple other advanced MRI techniques are based on DWI. Many of them focus on modeling diffusion in different tissue environments to disentangle the signal from “hindered, restricted, and isotropic compartments” (195). The hypothesis is that these compartments reflect actual tissue compartments, such as the extra-axonal or extracellular space (hindered diffusion), intra-cellular, or intra-axonal space (restricted), and fluid, such as edema or cerebrospinal fluid (isotropic). Many of these models, such as the NODDI technique, are designed based on assumptions in healthy white matter (123). Therefore, their use may be limited in glioblastomas. The RSI model (124) offers parametric indices to be calculated in a similar way to NODDI, such as with neurite density and dispersion indices, but RSI does allow more flexibility that may make it more appropriate for tumor imaging. While the tissue in RSI is modeled as cylinders, similar to other DWI-microstructure techniques targeted at white matter, the relatively large spectrum of size and orientation modeled makes it sensitive to a large range of geometries beyond the cylindrical shape (124). In addition, removal of the isotropic signal contribution can aid in disentangling enhancing tumor volume from edema. Disentangling the isotropic compartment is also of interest in the FWI technique. Here, a modeling approach that is initialized with a constant diffusivity of free water can be used to create maps of free water as well as produce a diffusion tensor that has the isotropic signal removed. Integration with the tensor approach lends its application to improving tractography results in the presence of a conspicuous amount of edema.

VERDICT is a model that infers tumor cell microstructure from DWI measurements (126). This model derives multiple compartments (intracellular, intravascular and extracellular–extravascular spaces) and has recently been used to investigate treatment response in animal models of glioma (196, 197). It has subsequently been adapted in human glioblastoma studies in treatment-naive patients (198). The VERDICT model assumes tumor cells are spherical and can model both cell radii and densities, making it an attractive option for the use in treatment response monitoring.

#### 3.9.2 Strengths and Weaknesses

Even though the advanced radiological imaging and multiparametric imaging approach is promising in the context of post-treatment related effects or specifically for pseudoprogression and radiation necrosis, efforts to establish uniform practices and protocols are again necessary. As in DWI and DTI, geometric distortions are also a challenge and there is no consensus on how many directions and which b-value shells are optimal with which particular microstructural technique. Other factors, such as the choice of TE and TR that is limited by either hardware or sequence, also add difficulty to the reproduction of results.

Many microstructural imaging approaches require lengthy protocols or uncommon scanner hardware options, such as parallel transmit or head coils with a large number of channels. In addition, the high b-values required often necessitate imaging close to the noise baseline, which requires lengthy averaging or noise modeling in post-processing, which is not a trivial challenge in clinical imaging.

#### 3.9.3 Evidence From Clinical Studies

**Figure 12:**
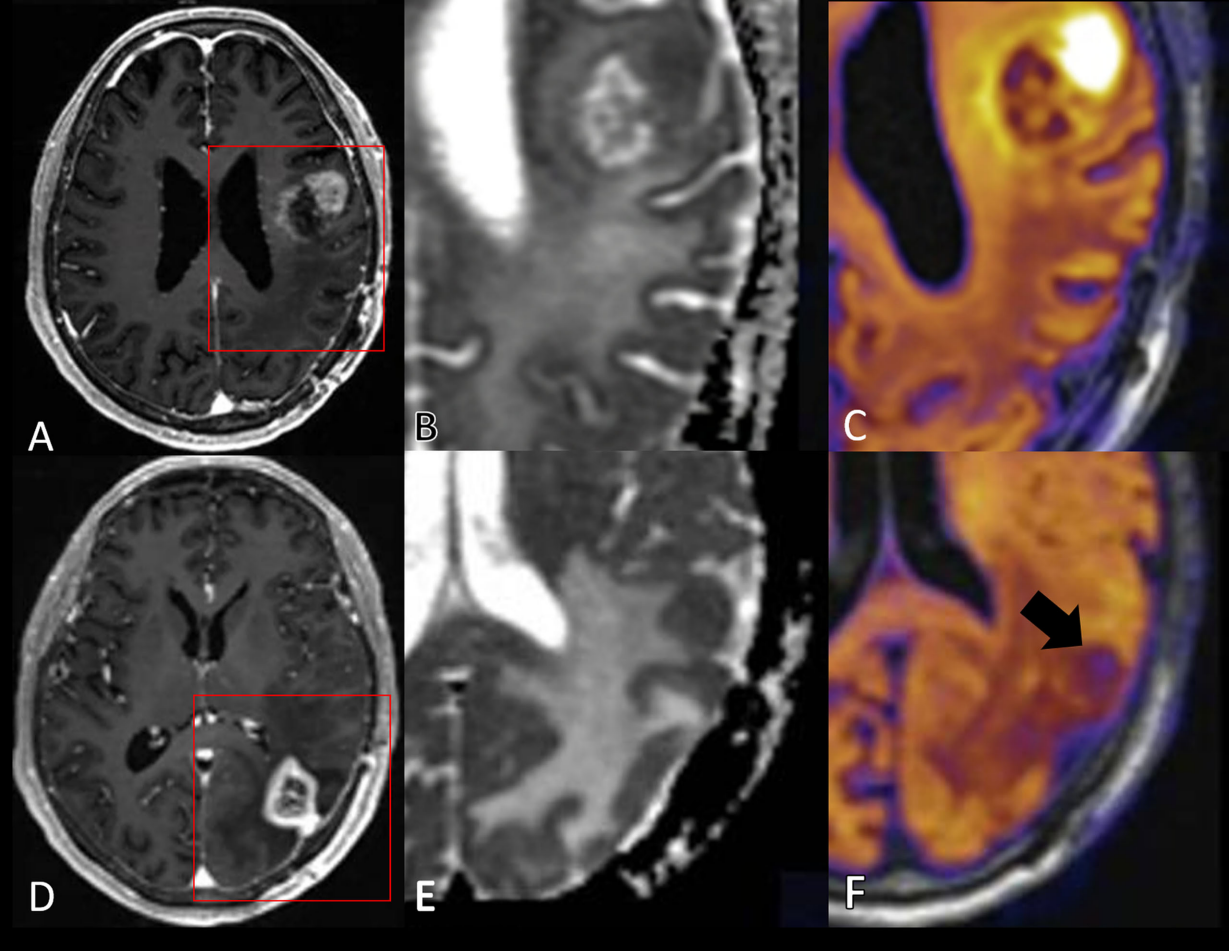
A glioblastoma in a 64-year-old man after chemoradiation and immunotherapy with both true progression **(A–C)** and pseudoprogression **(D–F)** observed in the same patient at different time points. Red squares in the T1-weighted post contrast images **(A, D)** illustrate the magnified regions on ADC images **(B, E)** and RSI cellularity map **(C, F)**. **(A–C)** An enhancing frontolateral area **(A)** confirmed to represent true progression is shown to have low ADC **(B)** with hyperintensity in the RSI cellularity map with more subtle ring hyperintensity around the central necrosis **(C)**. Left parietal pseudoprogression in an enhancing solid focus **(D)** was shown to be hypointense on the RSI cellularity map **(F)**. The ADC map is not decisive here **(E)**. Adapted with kind permission by the authors (204).

The evidence in clinical studies of brain tumor treatment response is very limited. However, FWI has most recently been used to successfully predict glioblastoma recurrence (199). Furthermore, RSI has been used to show treatment effects (135, 200, 201) as well as investigating overall and progression-free survival in gliomas (202). NODDI has been used together with serial DTI imaging to observe tumor changes up to two months before recurrence (203). For completeness in describing these emerging techniques, it is also noted that RSI was used to differentiate true progression from immune-mediated pseudoprogression (**Figure 12**) in a proof-of-concept case study. A 64-year-old man had follow-up imaging over several months. He developed pathologically proven pseudoprogression as well as a secondary primary lesion in a separate location. The authors found that RSI was able to differentiate true progression from pseudoprogression while ADC could not (204). However, whilst RSI is supported by marginally more clinical evidence than these other emerging techniques, it has yet to be adopted in large-scale glioblastoma research studies, thereby allowing the strengths of the technique to be demonstrated.

## 4 Discussion

Contemporaneous, accurate, and reliable monitoring biomarkers are required for high-grade glioma treatment response assessment as the use of conventional structural MRI protocols is limited by important challenges. The current evidence regarding the potential for monitoring biomarkers based on advanced MRI techniques is reviewed, and the individual modalities of perfusion, permeability, and microstructural imaging are described (these will be complemented by modalities related to metabolism and/or chemical composition discussed in Part 2). Considerable developments have been made in advanced MRI methodology. Although some techniques have evolved and matured over three decades, several new state-of-the-art methods are poised to contribute to the imaging armamentarium. However, limitations remain for all techniques. Good-quality evidence of clinical diagnostic accuracy is typically lacking. Clinical implementation of standardized tools generally remains challenging, and some recent techniques are in their infancy. The readiness of individual modalities in terms of technical validation, clinical evidence, acceptance and implementation are summarized in Part 2.

High-grade glioma vasculature exhibits increased perfusion, blood volume, and permeability compared with normal brain tissue. Measures of CBV derived from DSC-MRI have consistently provided information about brain tumor growth and response to treatment; it is the most clinically validated advanced technique. Clinical studies have proven the potential of DCE-MRI for distinguishing post-treatment related effects from recurrence, but the optimal acquisition protocol, mode of analysis, and parameter of highest diagnostic value and optimal cut-off points remain to be established. These same challenges are present to some extent with other perfusion techniques. ASL techniques do not require the injection of a contrast agent, and fast repeated measurements can be performed. Moreover, the absence of potential gadolinium deposition effects allows widespread use in pediatric patients and those with impaired renal function. Although the current review is focused on adult high-grade glioma, several studies have shown the utility of ASL perfusion imaging in pediatric brain tumors (205). DWI, ADC, DTI, DKI, IVIM, and other microstructural modeling approaches also allow treatment response assessment; more robust data is required to validate these techniques alone or when applied to post-processing methodologies. (Post-processing methodologies involving the combination of MRI approaches [multiparametric imaging] or machine learning are summarized in Part 2). Finally, for all parametric imaging techniques, the clinical application of cutoffs based on the literature is complicated by differences in how a ROI is defined and what particular descriptive statistic value (maximum, mean, etc.) within the ROI is used. Using a fixed image analysis approach, a multi-center study did show that excellent agreement of cut-offs between sites and platforms may be achieved (57). Nevertheless, until methods are standardized, generally accepted cut-off values may be difficult to apply in the clinical setting. Alternatively, clinical departments may rely on replicating approaches for generation and analysis of parametric images described in individual studies reporting the cut-off used.

In conclusion, considerable progress has been made in the development of advanced MRI monitoring biomarkers. As we approach the mid-twenty-first century, the techniques will have matured and serve as robust clinic-ready assays for treatment response assessment in personalized medicine regimens, which themselves will evolve in parallel. For now, more research and collaboration are needed to provide standardized and evidenced-based tools for this uncommon but disproportionately devastating disease.

## Author Contributions

All authors have contributed to the conception of the two parts of the article, revised them critically and approved the submitted versions. Authors TB and OH served as overall editors. The individual sections were drafted by: Introduction (TB), DSC-MRI (KS and MÁ-T), DCE-MRI (OH), ASL (PF and VK), diffusion techniques (RN, FR, and VK), spectroscopy (ECW and GH), CEST (EAHW), multiparametric imaging (OH), clinical readiness (OH), radiomics (TB), discussion (TB). All authors contributed to the article and approved the submitted version.

## Funding

This publication is part of the COST Action CA18206 Glioma MR Imaging 2.0 (www.glimr.eu), supported by COST (European Cooperation in Science and Technology), www.cost.eu. GliMR provided travel and accommodation for members who had travelled to early networking meetings. No participants were remunerated for their contribution. Funding support for KS: National Institute of Health/National Cancer Institute R01 CA255123, U01 CA176110, UG3 CA247606, Medical College of Wisconsin Cancer Center; GH: Austrian Science Fund grant KLI-646; ECW: The Dutch Research Council (NWO) Talent Programme Veni: 18144; EAHW: The Dutch Research Council (NWO) Talent Programme Veni: 91619121; PF: The Portuguese Foundation for Science and Technology (FCT) Grant UIDB/50009/2020; RN: Babes-Bolyai University, Grant GTC No. 35277/18.11.2020. TB was supported by the Wellcome/EPSRC Centre for Medical Engineering [WT 203148/Z/16/Z]. MÁ-T was supported by the ALBATROSS project (National Plan for Scientific and Technical Research and Innovation 2017-2020 and DPI2016-80054-R (Programa Estatal de Promoción del Talento y su Empleabilidad en I+D+i).

## Conflict of Interest

Author KS has ownership interest in IQ-AI Ltd and financial interest in Imaging Biometrics LLC. Author TB has participated in a speaker’s bureau for AbbVie and Siemens Healthineers.

The remaining authors declare that the research was conducted in the absence of any commercial or financial relationships that could be construed as a potential conflict of interest.

## Publisher’s Note

All claims expressed in this article are solely those of the authors and do not necessarily represent those of their affiliated organizations, or those of the publisher, the editors and the reviewers. Any product that may be evaluated in this article, or claim that may be made by its manufacturer, is not guaranteed or endorsed by the publisher.
