# Supplementary material for: High-Grade Glioma Treatment Response Monitoring Biomarkers: A Position Statement on the Evidence Supporting the Use of Advanced MRI Techniques in the Clinic, and the Latest Bench-to-Bedside Developments. Part 1: Perfusion and Diffusion Techniques
Source: Front Oncol. 2022 Mar 3;12:810263. doi: 10.3389/fonc.2022.810263 (PMC8961422; doi:10.3389/fonc.2022.810263)
Supplement: Supplementary file 1 [file DataSheet_1.zip › Supplementary Material Part 1.DOCX]

Supplementary Material: Part 1

# Supplementary Tables

**Table S1.** MEDLINE, EMBASE, and Cochrane Register search strategies. Informed by the Preferred Reporting Items for Systematic Reviews and Meta-Analysis: Diagnostic Test Accuracy (PRISMA-DTA) (1) and informed by Cochrane review methodology with emphasis on developing criteria for including and searching for studies (2,3).

**Search strategy and selection criteria**

Recommendations for a sensitive search with low precision—with subject headings with exploded terms, and with no language restrictions—were followed (2). Search terms were used in MEDLINE, EMBASE, and the Cochrane Register to describe each concept for original research articles published between June 2015 and June 2021. A search for preprints and other non-peer-reviewed material was not carried out.

| MEDLINE (OVID). PubMed was included.  The search strategy for Title/Abstract terms used a combination of subject headings (MeSH terms) and keywords:  Database: Ovid MEDLINE(R)  Search strategy:  1 exp Glioblastoma/  2 high grade glioma.mp.  3 pseudoprogression.mp.  4 imaging.mp.  5 exp Magnetic Resonance Imaging/ or MRI.mp.  6 pet.mp.  7 exp Positron-Emission Tomography/  8 1 or 2 or 3  9 4 or 5 or 6 or 7  10 8 and 9 |
| --- |
| EMBASE (OVID).  Subject headings and keywords:  Database: Embase  Search strategy:  1 exp Glioblastoma/  2 high grade glioma.mp.  3 pseudoprogression.mp.  4 exp multiparametric magnetic resonance imaging/ or exp imaging/ or exp nuclear magnetic resonance imaging/  5 magnetic resonance imaging.mp.  6 MRI.mp.  7 PET.mp. or exp positron emission tomography/  8 1 or 2 or 3  9 4 or 5 or 6 or 7  10 8 and 9  11 limit 10 to exclude medline journals |
| Cochrane Register.  Epistemonikos review database included, protocols included, CENTRAL (Cochrane central register of controlled trials included which includes <https://www.ebscohost.com/nursing/products/cinahl-databases>, <https://clinicaltrials.gov>, <https://www.who.int/ictrp/en/>).  Subject headings and keywords:  #1 MeSH descriptor: [Glioblastoma] explode all trees  #2 high grade glioma  #3 pseudoprogression  #4 imaging  #5 MeSH descriptor: [Magnetic Resonance Imaging] explode all trees  #6 MRI  #7 PET  #8 MeSH descriptor: [Positron-Emission Tomography] explode all trees  #9 {OR #1-#3}  #10 {OR #4-#8}  **#11 {AND #9-#10}** |
| Health Technology Assessment. <https://database.inahta.org/>  Subject headings and keywords:  (("Glioblastoma"[mh]) OR (high grade glioma) OR (pseudoprogression))  No results. |

**Table S2.** Overview of DCE studies of post-treatment diagnostic accuracy

| Study | Type | Other Modalities | n | WHO  Grade | Progression  compared to: | Frequency progressive  (n/n) | Pathology verified (n/n) | Analysis | Parameters | Best Parameter | Sensitivity | Specificity |
| --- | --- | --- | --- | --- | --- | --- | --- | --- | --- | --- | --- | --- |
| Bisdas 2011 (4) | Pro |  | 18 | III/IV | Radiation injury | 12/18 | 5/18 | Tofts + MF | V_e_, V_ep_, K^trans^, iAUC | K^trans^ > 0.19 min^-1^ | 100 | 83 |
| Narang 2011 (5) | Retro |  | 29  (22) | II-IV+MET  (HGG) | TIN | 20/29  (15/22) | 20/29 | MF | slope, iAUC | MSIVP (max slope) | 90  (100) | 100  (100) |
| Seeger 2013 (6) | Retro | ASL, DSC, MRS, ASL | 40 | III/IV | Stable disease | 23/40 | 0/40 | Tofts | V_e_, K^trans^ | max K^trans^ > 0.075 | 82 | 80 |
| Suh 2013 (7) | Retro |  | 79 | II-IV | PSP | 42/79 | 24/79 | MF | Various AUCR | mAURRh > 0.31 | 90 | 83 |
| Larsen 2013 (8) | Pro |  | 14 | II-IV | RN | 11/14 | 9/14 | Patlak | CBV, Ki, CBF (MF deconv) | absolute CBV > 2 ml/100g | 100 | 100 |
| Chung 2013 (9) | Retro |  | 57 | IV | RN | 32/57 | 5757 | MF | iAUC30, 60, 120, AUCR | mAUCRh > 0.23 | 94 | 88 |
| Shin 2014 (10) | Retro | DSC | 31 | II-IV | PTRE | 19/31 | 24/31 | Tofts + MF | K^trans^ ratio , IAUC ratio | relative K^trans^ > 2.1 | 61 | 80 |
| Kim 2014 (11) | Retro | DSC, DWI | 169 | IV | RN | 87/169 | 87/169 | MF | iAUC30, 60, 120 | iAUC30 > 15.8/15.2 combined w. T1CE+DWI | 91/92^a^ | 87/84^a^ |
| Yun 2015 (12) | Pro |  | 33 | IV | PSP | 17/33 | 0/33 | Ext Tofts | K^trans^, V_e_, V_p_ | 10p K^trans^ > 0.1 min^-1^ | 82 | 75 |
| Thomas 2015 (13) | Retro |  | 37 | IV | PSP | 24/37 | 0/37 | Ext Tofts | V_p_, K^trans^ | 90p relative V_p_ >3.9 | 92 | 85 |
| Hamilton 2015 (14) | Retro |  | 24 | II-IV | PTRE | 15/24 | 24/24 | Ext Tofts + MF | K^trans^, V_e_, V_p_, AUC | AUC (delayed short) > 20% | 93 | 78 |
| Park 2015 (15) | Retro | DSC, DWI | 108^b^  54^c^ | IV | PSP | 45/108  23/54 | 25/108  11/54 | MF | iAUC30 | iAUC > 19.4/19.9 | 82/84^a^  (78/83) | 81/82^a^  (81/84) |
| Heo 2015 (16) | Retro | DWI | 45 | GBM | PTRE | 20/45 | 9/45 | MF | iAUC90 | iAUC90, litt cut-off | 80/70^a^ | 72/74^a^ |
| Hatzaglou 2016 (17) | Pro | FDG | 53 (56^d^) | II-IV  +MET | “Radiation injury” | 38/55 | 14/55 | Ext Tofts | K^trans^, V_e_, V_p_, K_ep_, AUPC | Relative V_p_ > 2.1 | 92 | 77 |
| Yoo 2017 (18) | Pro |  | 24 | IV | RN | 16/24 | 0/24 | Ext Tofts | K^trans^, V_e_, V_p_ | Mean V_e_ < 0.87 | 100 | 63 |
| Zakhari 2018 (19) | Pro | DSC | 66 (68^d^) | III-IV | RN | 37(3)/68 | 43/68 | Ext Tofts + MF | K^trans^, V_p_, AUC | Relative V_p_ > 10.3 | 29 | 100 |
| Nael 2018 (20) | Retro | DSC, ADC | 46 | IV | RN | 34/46 | 36/46 | Ext Tofts | K^trans^ | K^trans^ > 0.1 min^-1^ | 68 | 83 |
| Seligman 2019 (21) | Retro | FDG | 41 | III-IV | PTRE | 32/41 | 12/41 | Ext Tofts | K^trans^, V_p_ | mean relative K^trans^ > 4.5 | 91 | 56 |
| Park 2021 (22) | Retro | APT, FA, DSC | 36 | II-IV | PTRE | 25/36 | 7/36 | Ext Toft | K^trans^, V_p_, V_e_, K_ep_ | n.r. | n.r | n.r |

Abbreviations: GBM = glioblastoma, HGG = high-grade gliomas, MET = metastases, MF = model free, n.r. = not reported, Pro = prospective, PSP = pseudoprogression, PTRE= post-treatment-related effects, Retro = retrospective, RN = radiation necrosis, TIN = “treatment-induced necrosis”, ^a^ result of 2 readers, ^b^ training cohort, ^c^ test cohort, ^d^ lesions. Note: WHO grade nomenclature for studies before 2021 WHO update.

**Table S3.** Overview of ASL studies of post-treatment diagnostic accuracy

| Study | Type | WHO Grade | n | Progression  compared to: | Frequency progressive (n/n) | Pathology verified (n/n) | Modality | Analysis | Sensitivity | Specificity |
| --- | --- | --- | --- | --- | --- | --- | --- | --- | --- | --- |
| Ozsunar 2010 (23) |  | II-IV | 30 | RN | 21/33 | 21/33 | PASL | nCBF (n=23) | 92-94 | 50 |
|  |  |  | (33 scans) |  |  |  |  | visual | 88 | 89 |
| Seeger 2013 (6) | Retro | HGG | 40 | PTRE | 23/40 | none | PASL | PASL | 54 | 85 |
| Choi 2013 (24) | Retro | GBM | 63 | PSP | 34/63 | 19/63 | PASL | PASL | 79 | 64 |
| Nyberg 2016 (25) | Retro | HGG | 22 | PTRE | all | all | 3D pCASL | aCBF | 100 | n.a. |
| Xu 2017 (26) | Pro | 18 HGG | 29 | PTRE | 17/29 | 12/29 | 3D pCASL | aCBF | 88 | 58 |
| Jovanovic 2017 (27) | Pro | GBM | 31 | PSP | 20/31 | none | 2D pCASL | nCBF | 100 | 74 |
| Razek 2018 (28) | Pro | HGG | 42 | PTRE | 24/42 | all | 3D pCASL | aCBF | 94 | 92 |
| Wang 2018 (29) | Retro | 55 HGG | 69 | PTRE | 35/69 | 37/69 | 3D pCASL | aCBF | 74 | 82 |
| Liu 2020 (30) | Pro | 25 HGG | 30 | PTRE | 16/30 |  | 3D pCASL | nCBF | 75 | 93 |
| Manning 2020 (31) | Retro | GBM | 32 | PSP | 25/32 | 9/32 | 3D pCASL | nCBF | 92 | 86 |
|  |  |  |  |  |  |  |  | aCBF | 100 | 88 |
| Pyatigorskaya 2021 (32) | Retro | HGG | 22 | RN | 18/22 | 8/22 | 3D pCASL | quant. | 90 | 75 |
|  |  |  |  |  |  |  |  | visual | 75 | 75 |
| Pellerin 2021 (33) | Pro | 48 HGG | 58 | PSP | 34/58 | N/K | 3D pCASL sTI | nCBF | 65 | 100 |

Abbreviations: GBM = glioblastoma, HGG = high-grade gliomas, Pro = prospective, PSP = pseudoprogression, PTRE = post-treatment-related effects, Retro = retrospective, RN = radiation necrosis. Note: WHO grade nomenclature for studies before 2021 WHO

# References

1. McInnes MDF, Moher D, Thombs BD, McGrath TA, Bossuyt PM, and the PRISMA-DTA Group, Clifford T, Cohen JF, Deeks JJ, Gatsonis C, et al. Preferred Reporting Items for a Systematic Review and Meta-analysis of Diagnostic Test Accuracy Studies: The PRISMA-DTA Statement. *JAMA* (2018) **319**: doi:10.1001/jama.2017.19163

2. de Vet HCV, Eisinga A, Riphagen II, Pewsner D. “Chapter 7: Searching for Studies,” in  *Cochrane Handbook for Systematic Reviews of Diagnostic Test Accuracy Version 0.4 [updated September 2008* (The Cochrane Collaboration).

3. Boosuyt P., Leeflang MM. “Chapter 6: Developing Criteria for Including Studies,” in *Cochrane Handbook for Systematic Reviews of Diagnostic Test Accuracy Version 0.4.*  (The Cochrane Collaboration.).

4. Bisdas S, Naegele T, Ritz R, Dimostheni A, Pfannenberg C, Reimold M, Koh TS, Ernemann U. Distinguishing Recurrent High-grade Gliomas from Radiation Injury. *Acad Radiol* (2011) **18**: doi:10.1016/j.acra.2011.01.018

5. Narang J, Jain R, Arbab AS, Mikkelsen T, Scarpace L, Rosenblum ML, Hearshen D, Babajani-Feremi A. Differentiating treatment-induced necrosis from recurrent/progressive brain tumor using nonmodel-based semiquantitative indices derived from dynamic contrast-enhanced T1-weighted MR perfusion. *Neuro Oncol* (2011) **13**: doi:10.1093/neuonc/nor075

6. Seeger A, Braun C, Skardelly M, Paulsen F, Schittenhelm J, Ernemann U, Bisdas S. Comparison of Three Different MR Perfusion Techniques and MR Spectroscopy for Multiparametric Assessment in Distinguishing Recurrent High-Grade Gliomas from Stable Disease. *Acad Radiol* (2013) **20**: doi:10.1016/j.acra.2013.09.003

7. Suh CH, Kim HS, Choi YJ, Kim N, Kim SJ. Prediction of Pseudoprogression in Patients with Glioblastomas Using the Initial and Final Area Under the Curves Ratio Derived from Dynamic Contrast-Enhanced T1-Weighted Perfusion MR Imaging. *Am J Neuroradiol* (2013) **34**: doi:10.3174/ajnr.A3634

8. Larsen VA, Simonsen HJ, Law I, Larsson HBW, Hansen AE. Evaluation of dynamic contrast-enhanced T1-weighted perfusion MRI in the differentiation of tumor recurrence from radiation necrosis. *Neuroradiology* (2013) **55**: doi:10.1007/s00234-012-1127-4

9. Chung WJ, Kim HS, Kim N, Choi CG, Kim SJ. Recurrent Glioblastoma: Optimum Area under the Curve Method Derived from Dynamic Contrast-enhanced T1-weighted Perfusion MR Imaging. *Radiology* (2013) **269**: doi:10.1148/radiol.13130016

10. Shin KE, Ahn KJ, Choi HS, Jung SL, Kim BS, Jeon SS, Hong YG. DCE and DSC MR perfusion imaging in the differentiation of recurrent tumour from treatment-related changes in patients with glioma. *Clin Radiol* (2014) **69**: doi:10.1016/j.crad.2014.01.016

11. Kim HS, Goh MJ, Kim N, Choi CG, Kim SJ, Kim JH. Which Combination of MR Imaging Modalities Is Best for Predicting Recurrent Glioblastoma? Study of Diagnostic Accuracy and Reproducibility. *Radiology* (2014) **273**: doi:10.1148/radiol.14132868

12. Yun TJ, Park C-K, Kim TM, Lee S-H, Kim J-H, Sohn C-H, Park S-H, Kim IH, Choi SH. Glioblastoma Treated with Concurrent Radiation Therapy and Temozolomide Chemotherapy: Differentiation of True Progression from Pseudoprogression with Quantitative Dynamic Contrast-enhanced MR Imaging. *Radiology* (2015) **274**: doi:10.1148/radiol.14132632

13. Thomas AA, Arevalo-Perez J, Kaley T, Lyo J, Peck KK, Shi W, Zhang Z, Young RJ. Dynamic contrast enhanced T1 MRI perfusion differentiates pseudoprogression from recurrent glioblastoma. *J Neurooncol* (2015) **125**: doi:10.1007/s11060-015-1893-z

14. Hamilton JD, Lin J, Ison C, Leeds NE, Jackson EF, Fuller GN, Ketonen L, Kumar AJ. Dynamic Contrast-Enhanced Perfusion Processing for Neuroradiologists: Model-Dependent Analysis May Not Be Necessary for Determining Recurrent High-Grade Glioma versus Treatment Effect. *Am J Neuroradiol* (2015) **36**: doi:10.3174/ajnr.A4190

15. Park JE, Kim HS, Goh MJ, Kim SJ, Kim JH. Pseudoprogression in Patients with Glioblastoma: Assessment by Using Volume-weighted Voxel-based Multiparametric Clustering of MR Imaging Data in an Independent Test Set. *Radiology* (2015) **275**: doi:10.1148/radiol.14141414

16. Heo YJ, Kim HS, Park JE, Choi C-G, Kim SJ. Uninterpretable Dynamic Susceptibility Contrast-Enhanced Perfusion MR Images in Patients with Post-Treatment Glioblastomas: Cross-Validation of Alternative Imaging Options. *PLoS One* (2015) **10**: doi:10.1371/journal.pone.0136380

17. Hatzoglou V, Yang TJ, Omuro A, Gavrilovic I, Ulaner G, Rubel J, Schneider T, Woo KM, Zhang Z, Peck KK, et al. A prospective trial of dynamic contrast-enhanced MRI perfusion and fluorine-18 FDG PET-CT in differentiating brain tumor progression from radiation injury after cranial irradiation. *Neuro Oncol* (2016) **18**: doi:10.1093/neuonc/nov301

18. Yoo R-E, Choi SH, Kim TM, Park C-K, Park S-H, Won J-K, Kim IH, Lee ST, Choi HJ, You S-H, et al. Dynamic contrast-enhanced MR imaging in predicting progression of enhancing lesions persisting after standard treatment in glioblastoma patients: a prospective study. *Eur Radiol* (2017) **27**: doi:10.1007/s00330-016-4692-9

19. Zakhari N, Taccone MS, Torres CH, Chakraborty S, Sinclair J, Woulfe J, Jansen GH, Cron GO, Thornhill RE, McInnes MDF, et al. Prospective comparative diagnostic accuracy evaluation of dynamic contrast-enhanced (DCE) vs. dynamic susceptibility contrast (DSC) MR perfusion in differentiating tumor recurrence from radiation necrosis in treated high-grade gliomas. *J Magn Reson Imaging* (2019) **50**: doi:10.1002/jmri.26621

20. Nael K, Bauer AH, Hormigo A, Lemole M, Germano IM, Puig J, Stea B. Multiparametric MRI for Differentiation of Radiation Necrosis From Recurrent Tumor in Patients With Treated Glioblastoma. *Am J Roentgenol* (2018) **210**: doi:10.2214/AJR.17.18003

21. Seligman L, Kovanlikaya I, Pisapia DJ, Naeger DM, Magge R, Fine HA, Chiang GC. Integrated PET-MRI for Glioma Surveillance: Perfusion-Metabolism Discordance Rate and Association With Molecular Profiling. *Am J Roentgenol* (2019) **212**: doi:10.2214/AJR.18.20531

22. Park YW, Ahn SS, Kim EH, Kang S-G, Chang JH, Kim SH, Zhou J, Lee S-K. Differentiation of recurrent diffuse glioma from treatment-induced change using amide proton transfer imaging: incremental value to diffusion and perfusion parameters. *Neuroradiology* (2021) **63**: doi:10.1007/s00234-020-02542-5

23. Ozsunar Y, Mullins ME, Kwong K, Hochberg FH, Ament C, Schaefer PW, Gonzalez RG, Lev MH. Glioma Recurrence Versus Radiation Necrosis? *Acad Radiol* (2010) **17**: doi:10.1016/j.acra.2009.10.024

24. Choi YJ, Kim HS, Jahng GH, Kim SJ, Suh DC. Pseudoprogression in patients with glioblastoma: Added value of arterial spin labeling to dynamic susceptibility contrast perfusion MR imaging. *Acta radiol* (2013) **54**:448–454. doi:10.1177/0284185112474916

25. Nyberg E, Honce J, Kleinschmidt-Demasters BK, Shukri B, Kreidler S, Nagae L. Arterial spin labeling: Pathologically proven superiority over conventional MRI for detection of high-grade glioma progression after treatment. *Neuroradiol J* (2016) **29**:377–383. doi:10.1177/1971400916665375

26. Xu Q, Liu Q, Ge H, Ge X, Wu J, Qu J, Xu K. Tumor recurrence versus treatment effects in glioma A comparative study of three dimensional pseudo-continuous arterial spin labeling and dynamic susceptibility contrast imaging. (2017) doi:10.1097/MD.0000000000009332

27. Jovanovic M, Radenkovic S, Stosic-Opincal T, Lavrnic S, Gavrilovic S-L, Lazovic-Popovic B, Soldatovic I, Maksimovic R. Differentiation between progression and pseudoprogression by arterial spin labeling MRI in patients with glioblastoma multiforme. *JBUON* (2017) **22**:1061–1067.

28. Razek AAKA, El-Serougy L, Abdelsalam M, Gaballa G, Talaat M. Differentiation of residual/recurrent gliomas from postradiation necrosis with arterial spin labeling and diffusion tensor magnetic resonance imaging-derived metrics. *Neuroradiology* (2018) **60**: doi:10.1007/s00234-017-1955-3

29. Wang YL, Chen S, Xiao HF, Li Y, Wang Y, Liu G, Lou X, Ma L. Differentiation between radiation-induced brain injury and glioma recurrence using 3D pCASL and dynamic susceptibility contrast-enhanced perfusion-weighted imaging. *Radiother Oncol* (2018) **129**:68–74. doi:10.1016/j.radonc.2018.01.009

30. Liu J, Li C, Chen Y, Lv X, Lv Y, Zhou J, Xi S, Dou W, Qian L, Zheng H, et al. Diagnostic performance of multiparametric MRI in the evaluation of treatment response in glioma patients at 3T. *J Magn Reson Imaging* (2020) **51**: doi:10.1002/jmri.26900

31. Manning P, Daghighi S, Rajaratnam MK, Parthiban S, Naeim Bahrami ·, Dale AM, Divya Bolar ·, Piccioni DE, Mcdonald CR, Farid N. Differentiation of progressive disease from pseudoprogression using 3D PCASL and DSC perfusion MRI in patients with glioblastoma. *J Neurooncol* (2020) **147**:681–690. doi:10.1007/s11060-020-03475-y

32. Pyatigorskaya N, Sgard B, Bertaux M, Yahia-Cherif L, Kas A. Can FDG-PET/MR help to overcome limitations of sequential MRI and PET-FDG for differential diagnosis between recurrence/progression and radionecrosis of high-grade gliomas? *J Neuroradiol* (2021) **48**: doi:10.1016/j.neurad.2020.08.003

33. Pellerin A, Khalifé M, Sanson M, Rozenblum-Beddok L, Bertaux M, Soret M, Galanaud D, Dormont D, Kas A, Pyatigorskaya N. Simultaneously acquired PET and ASL imaging biomarkers may be helpful in differentiating progression from pseudo-progression in treated gliomas. *Eur Radiol 2021* (2021)1–11. doi:10.1007/S00330-021-07732-0
